# Switchable pathways of multicomponent heterocyclizations of 5-amino-1,2,4-triazoles with salicylaldehydes and pyruvic acid

**DOI:** 10.3762/bjoc.21.158

**Published:** 2025-10-08

**Authors:** Yana I Sakhno, Oleksander V Buravov, Kostyantyn Yu Yurkov, Anastasia Yu Andryushchenko, Svitlana V Shishkina, Valentyn A Chebanov

**Affiliations:** 1 Institute of Functional Materials Chemistry, State Scientific Institution “Institute for Single Crystals” of National Academy of Sciences of Ukraine, Nauky Ave., 60, 61072, Kharkiv, Ukraine; 2 Enamine Ltd., Winston Churchill Street 78, Kyiv 02094, Ukraine; 3 Faculty of Chemistry, V. N. Karazin Kharkiv National University, Svobody sq., 4, 61022, Kharkiv, Ukraine

**Keywords:** 5-amino-1,2,4-triazole, heterocyclization, multicomponent reaction, salicylaldehyde, ultrasonication

## Abstract

Switchable multicomponent reactions involving 3-substituted-5-amino-1,2,4-triazoles, pyruvic acid, and salicylaldehydes were studied under different conditions. Upon conventional heating, benzotriazolooxadiazocine-5-carboxylic acids were formed in the three-component reactions as single reaction products. Upon ultrasonic activation or mechanical stirring at room temperature, the multicomponent reaction of the same starting materials led to the formation of only tetrahydrotriazolopyrimidine derivatives.

## Introduction

Multicomponent reactions (MCRs) are a powerful tool for the formation of heterocyclic systems in a minimal number of steps and offer several ways to vary substituents and they are widely used in drug discovery and development, as well as in diversity-oriented synthesis [1–4]. In addition, the application of condition-based divergence strategy [5] and the concept of multicomponent-switched reactions [6] allows to tune the selectivity of MCRs and direct them into several different pathways. That is why the study of multicomponent heterocyclizations of aromatic aldehydes, compounds with an active methylene group and aminoazoles, for example with 3-amino-1,2,4-triazoles, is important because the formation of different chemotypes of final heterocyclic compounds are possible depending on the structure of the reagents, the solvents and the catalysts, and type of activation methods [7–9].

MCRs of aminotriazoles, methylene-active compounds, and substituted salicylaldehydes are particularly interesting because of the possibility of additional reactions and post-cyclizations involving the *o*-hydroxy group. It was demonstrated [10–12] that the multicomponent reaction of substituted 3-amino-1,2,4-triazoles, various salicylaldehydes, and acetone depending on the conditions, can either be limited to a Biginelli condensation with the formation of hydroxytetrahydropyrimidines or proceed with further post-cyclization to form oxygen-bridged triazolobenzoxadiazocine derivatives. Furthermore, a multicomponent synthesis of oxygen-bridged pyrimidine systems also was described in several other publications [13–15]. On the other hand, three-component reactions of aminoazoles, salicylaldehyde, and esters (or amides) of acetoacetic acid with isolation of other types of heterocylic compounds were described in some papers [16–17].

**Scheme 1:**
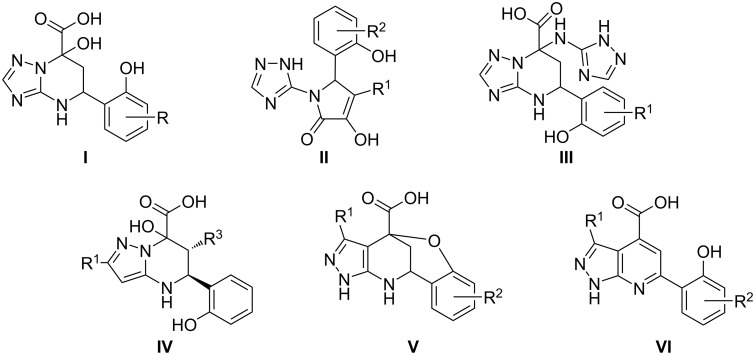
Diversity of heterocyclization products from reaction of aminoazoles with salicylaldehydes, and pyruvic acids.

Our early works were devoted to the study of the reactions of aminoazoles, pyruvic acid and its derivatives with salicylaldehydes and it was found that depending on the conditions (reaction time, temperature, and method of process activation, in particular ultrasound and microwave irradiation), different types of heterocycles **I**–**VI** were formed (Scheme 1) [8,18–19].

It is known that substituents in aminoazoles can significantly influence their behavior in multicomponent heterocyclization reactions [4,8,20–21]. However, the reactions of 5-amino-1,2,4-triazoles containing substituents in position 3, which can also affect their chemical behaviour, with pyruvic acid, and salicylaldehydes remain undescribed. The present paper is intended to fill this gap and complete the description of this type of switchable multicomponent reactions.

## Results and Discussion

**Scheme 2:**
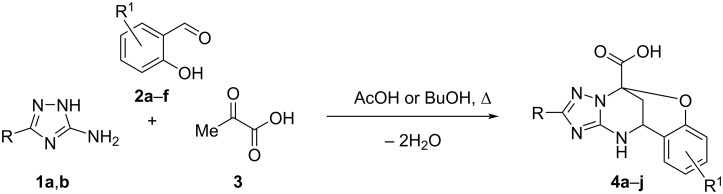
MCRs of 3-amino-5-methylthio-1,2,4-triazole (**1a**) and 3-amino-5-methoxy-1,2,4-triazole (**1b**) with salicylaldehydes **2a**–**f**, and pyruvic acid (**3**).

Therefore, the MCRs between some 3-substituted 5-amino-1,2,4-triazoles, salicylaldehydes, and pyruvic acid were studied under different reaction conditions. In particular, it was found that the three-component reaction of an equimolar mixture of 5-amino-3-methylthio(methoxy)-1,2,4-triazole **1a,b**, salicylaldehydes **2a**–**f**, and pyruvic acid (**3**) under conventional heating at reflux in acetic acid for 3 h for aminotriazole **1a** or in *n*-BuOH for 7 h in case of aminotriazole **1b** led to the formation of oxygen-bridged 2-(methylthio or methoxy)-11,12-dihydro-5*H*-5,11-methanobenzo[*g*][1,2,4]triazolo[1,5-*c*][1,3,5]oxadiazocine-5-carboxylic acids **4a**–**j** (Scheme 2, Table 1). It should be noted that this MCR involving unsubstituted 5-amino-1*H*-1,2,4-triazole under various conditions never proceeds towards the formation of oxygen-bridged compounds [18]. Thus, there is indeed a significant influence of the substituent in position 3 of 5-amino-1*H*-1,2,4-triazoles on their chemical behavior in this MCR.

**Table 1:** Synthesis of compounds **4a**–**j**.

Entry	Starting materials				Reaction time	Product	Yield
	Compd.	R	Compd.	R^1^	(hours)		(%)

1	**1a**	SCH_3_	**2a**	H	3	**4a**	45
2	**1a**	SCH_3_	**2b**	3-CH_3_O	3	**4b**	60
3	**1a**	SCH_3_	**2c**	5-Br	3	**4c**	42
4	**1a**	SCH_3_	**2d**	5-NO_2_	3	**4d**	35
5	**1a**	SCH_3_	**2e**	5-F	3	**4e**	32
6	**1a**	SCH_3_	**2f**	5-Cl	3	**4f**	42
7	**1b**	OCH_3_	**2b**	3-CH_3_O	7	**4g**	46
8	**1b**	OCH_3_	**2c**	5-Br	7	**4h**	41
9	**1b**	OCH_3_	**2e**	5-F	7	**4i**	35
10	**1b**	OCH_3_	**2f**	5-Cl	7	**4j**	68

In the case of 3-carbomethoxy- and 3-(trifluoromethyl)-5-amino-1,2,4-triazoles the treatment with pyruvic acid and salicylaldehydes under thermal heating in different solvents (AcOH, DMF, *n*-butanol), instead of oxygen-bridged compounds gave a mixture of unidentified products with trace amounts of Schiff bases between amino-1,2,4-triazole and salicylaldehyde as well as with impurities of the reagents. This is most likely due to the electron-withdrawing nature of the substituent at C(3) in these 5-amino-1,2,4-triazoles that do not favor the proceeding of the MCR in contrast to 5-aminotriazoles **1a,b** having pronounced electron-donating substituents (methylthio- and methoxy groups).

**Scheme 3:**
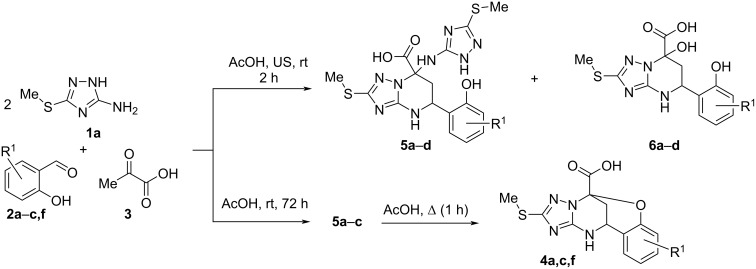
MCRs of 3-amino-5-methylthio-1,2,4-triazole (**1a**), salicylaldehydes **2a**–**c**,**f**, and pyruvic acid (**3**) under different conditions.

On the other hand, the MCR of the reagents **1a**, **2a**–**c** and **3** under ultrasonic irradiation for 2 h at room temperature (25 °C) (Scheme 3) afforded a mixture of 5-(2-hydroxyphenyl)-2-(methylthio)-7-((3-(methylthio)-4*H*-1,2,4-triazol-5-yl)amino)-4,5,6,7-tetrahydro[1,2,4]triazolo[1,5-*a*]pyrimidine-7-carboxylic acids **5a**–**d** and 5-(2-hydroxyphenyl)-7-hydroxy-2-(methylthio)-4,5,6,7-tetrahydro[1,2,4]triazolo[1,5-*a*]pyrimidine-7-carboxylic acids **6a**–**d** (in a ratio of ca. 80:20 for compounds **5a**–**c** and **6a**–**c**; and 15:85 for compounds **5d** and **6d**). Unfortunately, all attempts to separate these mixtures were unsuccessful.

However, repeating the reaction under mechanical stirring at room temperature for 72 h yielded tetrahydrotriazolopyrimidine-7-carboxylic acids **5a**–**c** as a mixture of two diastereomers (Scheme 3, Table 2). In the case of aldehyde **2b**, the MCR always led to the formation of a mixture of products **5d** and **6d**. Attempts to synthesize **5d** and **6d** as individual compounds under various conditions were unsuccessful.

**Table 2:** Synthesis of compounds **5a**–**c**.

Entry	Starting materials		Reaction time	Temperature regime	Product	Yield
	Compd.	R^1^	(hours)	Method		(%)

1	**2a**	H	72	mech. stir, rt	**5a**	58
2	**2c**	5-Br	72	mech. stir, rt	**5b**	69
3	**2f**	5-Cl	72	mech. stir, rt	**5c**	67

In addition, it was found that compounds **5** can be converted into oxygen-bridged heterocycles **4** after 1 hour of conventional heating at reflux in acetic acid (Scheme 3). In contrast, all attempts to transform 7-triazolylamino derivatives of pyrimidinecarboxylic acids **5** into their 7-hydroxy derivatives **6** were unsuccessful. It also failed to perform the reverse conversion. The same situation was observed for similar compounds based on 5-amino-1*H*-pyrazole-4-carbonitrile [22].

It should be noted that diazocines **4** were stable and did not undergo transformations when heated or irradiated with ultrasound. This may indicate that pyrimidine-7-carboxylic acids **5** and **6** are formed in parallel processes under kinetic control of the reaction, while oxygen-bridged heterocycles **4** are formed under thermodynamic control.

**Figure 1:**
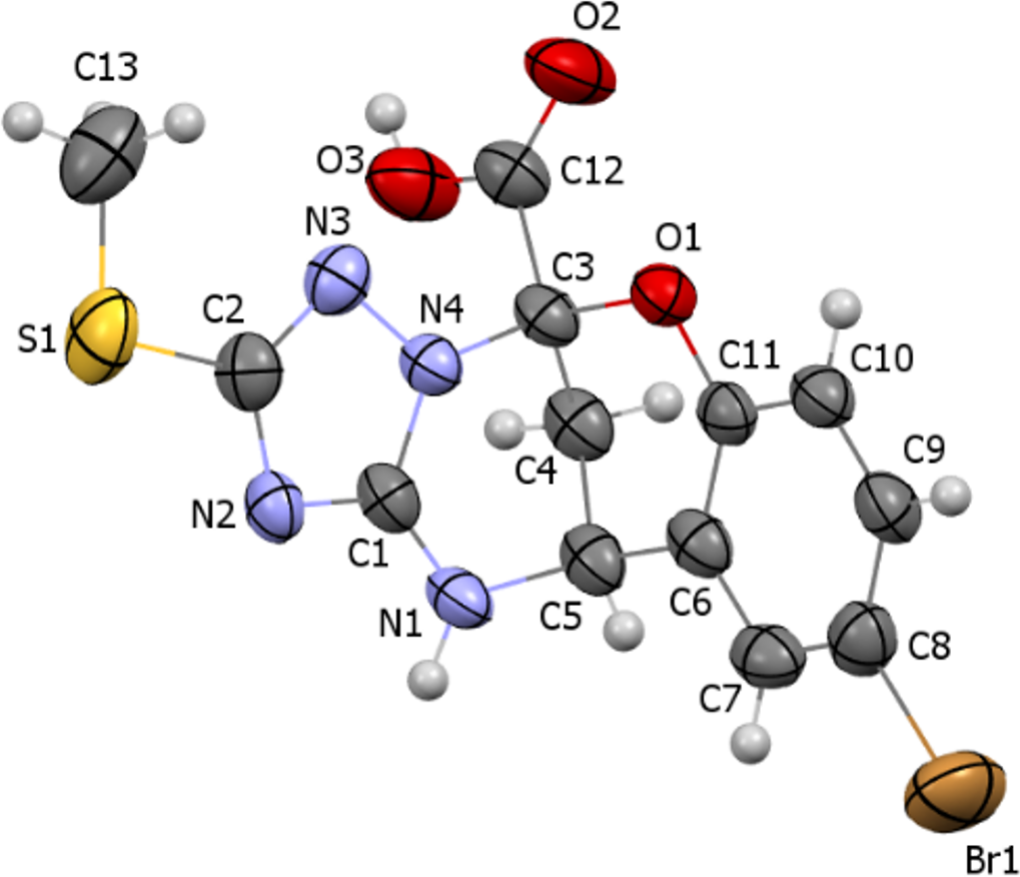
Molecular structure of compound **4c** according to X-ray diffraction data. Thermal ellipsoids are shown at 50% probability level.

The purity and structure of compounds **4** and **5** were established by elemental analysis, mass spectrometry, ^1^H and ^13^C NMR spectroscopy, and X-ray diffraction study (Figure 1).

For example, the ^1^H NMR spectra of products **4a**–**f** exhibit signals for proton of the pyrimidine NH group (8.38–8.53 ppm), signals for aromatic protons (6.58–8.34 ppm), multiplet for the proton of the CH group at 4.64–4.92 ppm, a singlet for the protons for the triazole SCH_3_ group at 2.37–2.38 ppm as well as signals for other substituents. The peaks of the proton of the carboxyl group is not recognized, probably due to exchange processes. The signals of the CH_2_ group in the range around δ = 2.5 ppm are not always clearly identifiable, as they partially or completely overlap with the remaining DMSO signal. Compounds **4g**–**j** are characterized by the following signals in the ^1^H NMR spectra: a proton singlet of the pyrimidine NH group at 8.30–8.31 ppm, a multiplet of aromatic protons in the region of 6.95–7.51 ppm, a proton multiplet of the CH group at 4.65–4.68 ppm and singlet for protons for the triazole CH_3_O group at 3.7 ppm.

The ^1^H NMR spectra of heterocyclic acids **5a**–**c** exhibited a broad singlet of proton of the carboxyl group at 11.88–14.02 ppm, a singlet of proton of the hydroxy group at 9.41–9.86 ppm, a singlet of proton of the pyrimidine NH group at 7.72–7.91 ppm, a singlet of proton of the triazolylamine NH group at 7.44–7.57 ppm, peaks for aromatic protons at 6.70–7.52 ppm, a multiplet of proton of the CH group at 5.0–5.19 ppm and two signals for diastereotopic protons of CH_2_ group (about 2.89–3.11 and 2.28–2.35 ppm) and protons for the triazole SCH_3_ group at 2.38–2.46 ppm. The doubling of some signals in the ^1^H NMR spectra probably indicates the formation of both possible diastereomers. The structure of compounds **6a**–**d** was confirmed from the ^1^H NMR spectra and the mass spectra of mixture by comparison with literature data for similar pyrimidines [17–18].

## Conclusion

In summary, the multicomponent reaction of 3-amino-5-methylthio(methoxy)-1,2,4-triazoles with salicylaldehydes, and pyruvic acid can be switched between two different directions using conventional thermal heating, mechanical stirring at room temperature and ultrasonication at room temperature. The treatment under reflux conditions leads to the formation of oxygen-bridged benzo[*g*][1,2,4]triazolo[1,5-*c*][1,3,5]oxadiazocine-5-carboxylic acids exclusively while the reaction under mechanical stirring gives 5-(2-hydroxyphenyl)-2-(methylthio)-7-((3-(methylthio)-4*H*-1,2,4-triazol-5-yl)amino)-4,5,6,7-tetrahydro[1,2,4]triazolo[1,5-*a*]pyrimidine-7-carboxylic acids. The reaction under ultrasonication at room temperature yields a mixture of the latter with 5-(2-hydroxyphenyl)-7-hydroxy-2-(methylthio)-4,5,6,7-tetrahydro[1,2,4]triazolo[1,5-*a*]pyrimidine-7-carboxylic acids.

## Supporting Information

File 1 Experimental procedures, product characterization, and copies of NMR spectra.

## Data Availability

Data generated and analyzed during this study is available from the corresponding author upon reasonable request.
