# Supplementary material for: Switchable pathways of multicomponent heterocyclizations of 5-amino-1,2,4-triazoles with salicylaldehydes and pyruvic acid
Source: Beilstein J Org Chem. 2025 Oct 8;21:2030–5. doi: 10.3762/bjoc.21.158 (PMC12522153; doi:10.3762/bjoc.21.158)
Supplement: File 1 — Experimental procedures, product characterization, and copies of NMR spectra. [file Beilstein_J_Org_Chem-21-2030-s001.pdf]

BEILSTEIN JOURNAL OF ORGANIC CHEMISTRY

Supporting Information

for

Switchable pathways of multicomponent heterocyclizations of 5-amino-1,2,4-triazoles with salicylaldehydes and pyruvic acid

Yana I. Sakhno, Oleksander V. Buravov, Kostyantyn Yu. Yurkov,
Anastasia Yu. Andryushchenko, Svitlana V. Shishkina and Valentyn A. Chebanov

Beilstein J. Org. Chem. **2025,** *21,* 2030–2035. doi:10.3762/bjoc.21.158

Experimental procedures, product characterization, and copies of NMR spectra

License and Terms: This is a supporting information file under the terms of the Creative Commons Attribution License (https://creativecommons.org/licenses/by/4.0). Please note that the reuse, redistribution and reproduction in particular requires that the author(s) and source are credited and that individual graphics may be subject to special legal provisions.
The license is subject to the *Beilstein Journal of Organic Chemistry* terms and conditions: (https://www.beilstein-journals.org/bjoc/terms)

Experimental section

Instrumentation and chemicals

^{1}H and ^{13}C NMR spectra were recorded on a Bruker Avance III, Bruker Avance DRX and Varian Unity INOVA spectrometers (400 and 100 MHz, respectively) in DMSO-d_6. Mass spectra were measured on LC/MSD Agilent 1100 and Shimadzu LCMS-2020. Elemental analysis was performed on a Euro Vector EA-3000. Melting points of all synthesized compounds were determined using OptiMelt MPA100 electronic melting point apparatus and are uncorrected.

Ultrasound-assisted experiments were carried out using a standard ultrasound bath (SELDI, Ukraine) with a working frequency of 44.2 kHz.

All 1,2,4-triazol-5-amines, 2-oxopropanoic acid, and salicylaldehydes were commercially available.

X-ray experimental part

The colourless crystals of compound **4c** ($C_{13}H_{11}BrN_4O_3S$, C_2H_6OS) are triclinic. At 173 K a = 5.8414(5), b = 12.2797(9), c = 14.2024(9) Å, α = 109.169(4)°, β = 91.976(5)°, γ = 95.085(5)°, V = 956.28(13) Å^3, M_r = 461.36, Z = 2, space group $P\bar{1}$, d_{calc}= 1.602 g/cm^3, μ(Mo K_α) = 2.396 mm^{-1}, F(000) = 468. Intensities of 14010 reflections (3360 independent, R_{int}=0.0573) were measured on a Bruker APEX II diffractometer (graphite monochromated Mo K_α radiation, CCD detector, φ- and ω-scaning, $2\Theta_{max}$ = 50°). The structure was solved by direct method using OLEX2 [1] package with SHELXT [2] and SHELXL modules [3]. The absorption correction was done using the 'multi-scan' method (T_{min} = 0.3880, T_{max} = 0.7454). Positions of the hydrogen atoms were located from electron density difference maps and refined using "riding" model with $U_{iso} = nU_{eq}$ (n = 1.5 for methyl groups and n = 1.2 for other hydrogen atoms) of the carrier atom. Hydrogen atom or the hydroxy group was refined using the isotropic approximation. Full-matrix least-squares refinement against F^2 in anisotropic approximation for non-hydrogen atoms using 3360 reflections was converged to wR_2 = 0.2488 (R_1 = 0.0718 for 1999 reflections with F>4σ(F), S = 1.038). The final atomic coordinates, and crystallographic data for molecule **4c** have been deposited to with the Cambridge Crystallographic Data Centre, 12 Union Road, CB2 1EZ, UK (fax: +44-1223-336033; e-mail: deposit@ccdc.cam.ac.uk) and are available on request quoting the deposition numbers CCDC 2418714).

General procedure for the synthesis of 2-(methylthio)-11,12-dihydro-5*H*-5,11-methanobenzo[*g*][1,2,4]triazolo[1,5-*c*][1,3,5]oxadiazocine-5-carboxylic acid and its derivatives 4a–f

A mixture of 5-amino-3-methylthio-1*H*-1,2,4-triazole (**1a**, 130 mg, 1 mmol), appropriate substituted salicylaldehyde **2a–c** (1 mmol) and pyruvic acid (**3**, 88 mg, 1 mmol) in 2 mL of acetic acid was heated at reflux for 3 h. After cooling the reaction mixture was left overnight at room temperature until a precipitate was formed, which was filtered off and vacuum-dried.

Procedure for the synthesis of compounds 4a,c,f from tetrahydrotriazolopyrimidine-7-carboxylic acid 5a–c

Compound **5** (1 mmol) was heated at reflux for 1 h in 2 mL of acetic acid. After cooling, the precipitate was filtered off and vacuum-dried.

Characterization data

2-(Methylthio)-11,12-dihydro-5*H*-5,11-methanobenzo[*g*][1,2,4]triazolo[1,5-*c*][1,3,5]oxa-diazocine-5-carboxylic acid (4a)

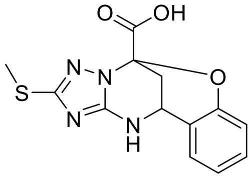

White solid (137 mg, 45%), mp 252–254°C (AcOH). ^{1}H NMR spectrum, (400 MHz, DMSO-*d*$_6$), δ, ppm (*J*, Hz): 2.38 (3H, s, CH_3S); 4.63-4.69 (1H, m, CH); 6.92-7.35 (4H, m, ArH); 8.38 (1H, s, NH). ^{13}C NMR spectrum, (100 MHz, DMSO-*d*$_6$), δ, ppm: 13.3; 29.8; 44.1; 82.0; 117.1; 122.1; 123.9; 129.9; 129.9; 150.0; 154.8; 158.9; 166.2. Mass spectrum *m/z* (ESI, %): 305 $[M+H]^+$ (100); 175 (21). Found, %: C 51.21; H 3.83; N 18.53. $C_{13}H_{12}N_4O_3S$. Calculated, %: C 51.31; H 3.97; N 18.41.

7-Methoxy-2-(methylthio)-11,12-dihydro-5*H*-5,11-methanobenzo[*g*][1,2,4]triazolo[1,5-*c*][1,3,5]-oxadiazocine-5-carboxylic acid (4b)

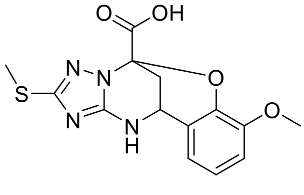

White solid (200 mg, 60%), mp 259–261°C (AcOH). ^{1}H NMR spectrum, (400 MHz, DMSO-*d*$_6$), δ, ppm (*J*, Hz): 2.38 (3H, s, CH_3S); 2.46-2.49 (2H, m, CH_2); 3.75 (3H, s, CH_3O); 4.62-4.65 (1H, m, CH); 6.87-6.98 (3H, m, ArH); 8.43 (1H, s, NH). ^{13}C NMR spectrum, (100 MHz, DMSO-*d*$_6$), δ, ppm: 13.2; 29.7; 44.0; 55.5; 81.8; 112.1; 121.1; 121.9; 124.4; 139.4; 148.3; 154.7; 158.8; 166.1. Mass spectrum *m/z* (ESI, %): 335 $[M+H]^+$ (100); 336 (17). Found, %: C 50.16; H 4.13; N 16.89. $C_{14}H_{14}N_4O_4S$. Calculated, %: C 50.29; H 4.22; N 16.76.

9-Bromo-2-(methylthio)-11,12-dihydro-5*H*-5,11-methanobenzo[*g*][1,2,4]triazolo[1,5-*c*][1,3,5]-oxadiazocine-5-carboxylic acid (4c)

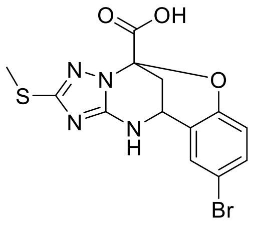

White solid (161 mg, 42%), mp 273–275°C (AcOH). ^{1}H NMR spectrum, (400 MHz, DMSO-d_6), δ, ppm (*J*, Hz): 2.37 (3H, s, CH_3S); 2.51-2.53 (2H, m, CH_2); 4.69-4.73 (1H, m, CH); 6.93-7.53 (3H, m, ArH); 8.42 (1H, s, NH). ^{13}C NMR spectrum, (100 MHz, DMSO-d_6), δ, ppm: 13.3; 29.3; 43.6; 82.0; 113.3; 119.5; 126.3; 132.3; 132.4; 149.3; 154.6; 159.1; 165.7. Mass spectrum *m/z* (ESI, %): 383 $[M+H]^+$ (65); 385 $[M+H]^+$ (55); 101 (7).
Found, %: C 40.59; H 2.78; N 14.56. $C_{13}H_{11}BrN_4O_3S$. Calculated, %: C 40.75; H 2.89; N 14.62.

2-(Methylthio)-9-nitro-11,12-dihydro-5*H*-5,11-methanobenzo[*g*][1,2,4]triazolo[1,5-*c*][1,3,5]-oxadiazocine-5-carboxylic acid (4d)

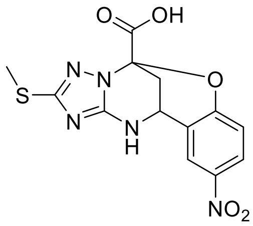

Beige solid (122 mg, 35%), mp 249–251°C (AcOH). ^{1}H NMR spectrum, (400 MHz, DMSO-d_6), δ, ppm (*J*, Hz): 2.37 (3H, s, CH_3S); 2.60-2.66 (2H, m, CH_2); 4.90-4.93 (1H, m, CH); 7.21-8.36 (3H, m, ArH); 8.53 (1H, s, NH). ^{13}C NMR spectrum, (100 MHz, DMSO-d_6), δ, ppm: 13.3; 29.1; 43.6; 82.5; 118.5; 125.0; 125.5; 126.1; 141.8; 154.5; 155.5; 159.6; 165.3. Mass spectrum *m/z* (ESI, %): 350 $[M+H]^+$ (100); 351 (15). Found, %: C 44.59; H 3.08; N 20.16. $C_{13}H_{11}N_5O_5S$. Calculated, %: C 44.70; H 3.17; N 20.05.

9-Fluoro-2-(methylthio)-11,12-dihydro-5*H*-5,11-methanobenzo[*g*][1,2,4]triazolo[1,5-*c*][1,3,5]oxadiazocine-5-carboxylic acid (4e)

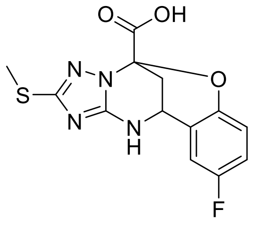

White solid (103 mg, 32%), mp 265–267°C (AcOH). ^{1}H NMR spectrum, (400 MHz, DMSO-d_6), δ, ppm (*J*, Hz): 2.37 (3H, s, CH_3S); 4.67-4.71 (1H, m, CH); 6.97- 7.20 (3H, m, ArH); 8.41 (1H, s, NH). ^{13}C NMR spectrum, (100 MHz, DMSO-d_6), δ, ppm: 13.3; 29.4; 44.0; 82.1; 115.6; 115.8; 116.7; 116.9; 118.7; 118.8; 125.2; 146.2; 154.7; 155.9; 157.8; 159.1; 165.9. Mass spectrum *m/z* (ESI, %): 323

[M+H]$^+$ (100); 324 (15); 101 (11). Found, %: C 48.31; H 3.39; N 17.49. $C_{13}H_{11}FN_4O_3S$. Calculated, %: C 48.44; H 3.44; N 17.38.

9-Chloro-2-(methylthio)-11,12-dihydro-5*H*-5,11-methanobenzo[*g*][1,2,4]triazolo[1,5-*c*][1,3,5]oxadiazocine-5-carboxylic acid (4f)

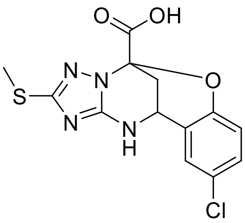

White solid (142 mg, 42%), mp 260–262°C (AcOH). ^{1}H NMR spectrum, (400 MHz, DMSO-*d*$_6$), δ, ppm (*J*, Hz): 2.38 (3H, s, CH_3S); 2.51-2.56 (2H, m, CH_2); 4.71-4.73 (1H, m, CH); 7.01-7.39 (3H, m, ArH); 8.41 (s, 1H, NH). ^{13}C NMR spectrum, (100 MHz, DMSO-*d*$_6$), δ, ppm: 13.2; 29.3; 43.7; 82.0; 119.1; 125.6; 125.8; 129.3; 129.6; 148.9; 154.6; 159.1; 165.7. Mass spectrum *m/z* (ESI, %): 339 [M+H]$^+$ (100); 341 (42). Found, %: C 45.96; H 3.18; N 16.63. $C_{13}H_{11}ClN_4O_3S$. Calculated, %: C 46.09; H 3.27; N 16.54.

General Procedure for the synthesis of 2-(methoxy)-11,12-dihydro-5*H*-5,11-methanobenzo[*g*][1,2,4]triazolo[1,5-*c*][1,3,5]oxadiazocine-5-carboxylic acid and its derivatives 4g–j

A mixture of 5-amino-3-methoxy-1*H*-1,2,4-triazole (**1b**, 114 mg, 1 mmol), appropriate substituted salicylaldehyde **2b,c,e,f** (1 mmol) pyruvic acid (**3**, 88 mg, 1 mmol) in 2 mL of *n*-BuOH was heated at reflux for 7 h. Then *n*-BuOH was evaporated from the reaction mixture under reduced pressure, then 2 mL acetone added to the residue and allowed to crystallize. The precipitate was filtered and dried.

2,7-Dimethoxy-11,12-dihydro-5*H*-5,11-methanobenzo[*g*][1,2,4]triazolo[1,5-*c*] [1,3,5]oxadiazocine-5-carboxylic acid (4g)

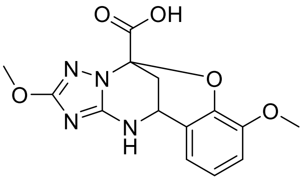

White solid (146 mg, 46%), mp 261–263°C (acetone). ^{1}H NMR spectrum, (400 MHz, DMSO-*d*$_6$), δ, ppm (*J*, Hz): 3.69 (3H, s, CH_3O); 3.75 (3H, s, CH_3O); 4.60 (1H, s, CH); 6.85-6.98 (3H, m, ArH); 8.34 (1H, s, NH). ^{13}C NMR spectrum, (100 MHz, DMSO-*d*$_6$), δ, ppm: 29.8; 43.8; 55.5; 55.7; 81.6; 112.1; 121.0; 121.7; 124.4; 139.6; 148.3; 153.6; 166.1; 166.1. Mass spectrum m/z (ESI, %): 319 [M+H]$^+$ (100); 320 (18). Found, %: C 52.72; H 4.30; N 17.56. $C_{14}H_{14}N_4O_5$. Calculated, %: C 52.83; H 4.43; N 17.60.

9-Bromo-2-methoxy-11,12-dihydro-5*H*-5,11-methanobenzo[*g*][1,2,4]triazolo[1,5-*c*][1,3,5]oxadiazocine-5-carboxylic acid (4h)

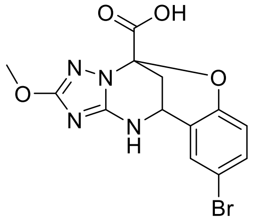

White solid (151 mg, 41%), mp 274–276°C (acetone). ^{1}H NMR spectrum, (400 MHz, DMSO-d_6), δ, ppm (*J*, Hz): 3.71 (3H, s, CH_3O); 4.65-4.68 (1H, m, CH); 6.94-7.51 (3H, m, ArH); 8.29 (1H, s, NH). ^{13}C NMR spectrum, (100 MHz, DMSO-d_6), δ, ppm: 29.4; 43.6; 55.8; 81.8; 113.2; 119.5; 126.4; 132.2; 132.4; 149.4; 153.7; 165.8; 166.2. Mass spectrum *m/z* (ESI, %): 367 [M+H]+ (99); 369 $[M+H]^+$ (100). Found, %: C 42.49; H 2.98; N 15.39. $C_{13}H_{11}BrN_4O_4$. Calculated, %: C 42.53; H 3.02; N 15.26.

9-Fluoro-2-methoxy-11,12-dihydro-5*H*-5,11-methanobenzo[*g*][1,2,4]triazolo[1,5-*c*][1,3,5]oxadiazocine-5-carboxylic acid (4i)

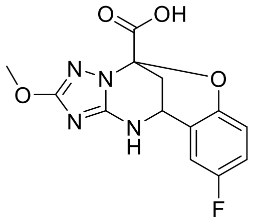

White solid (107 mg, 35%), mp 256–258°C (acetone). ^{1}H NMR spectrum, (400 MHz, DMSO-d_6), δ, ppm (*J*, Hz): 3.70 (3H, s, CH_3O); 4.63-4.66 (1H, m, CH); 6.98-7.15 (3H, m, ArH), 8.31 (1H, s, NH). ^{13}C NMR spectrum, (100 MHz, DMSO-d_6), δ, ppm: 30.0; 43.8; 55.7; 81.8; 115.5; 115.7; 116.5; 118.6; 118.7; 125.2; 146.3; 153.7; 155.9; 157.6; 165.9; 166.2. Mass spectrum *m/z* (ESI, %): 307 $[M+H]^+$ (100); 308 (21). Found, %: C 50.79; H 3.51; N 18.36. $C_{13}H_{11}FN_4O_4$. Calculated, %: C 50.98; H 3.62; N 18.29.

9-Chloro-2-methoxy-11,12-dihydro-5*H*-5,11-methanobenzo[*g*][1,2,4]triazolo[1,5-*c*][1,3,5]oxadiazocine-5-carboxylic acid (4j)

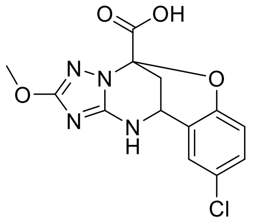

White solid (219 mg, 68%), mp 270–272°C (acetone). ^{1}H NMR spectrum, (400 MHz, DMSO-d_6), δ, ppm (*J*, Hz): 3.71 (3H, s, CH_3O); 4.68 (1H, s, CH); 6.99-7.38 (3H, m, ArH); 8.29 (1H, s, NH). ^{13}C NMR spectrum, (100 MHz, DMSO-d_6), δ, ppm: 29.5; 43.7; 55.8; 81.9; 119.1; 125.5; 125.9; 129.3; 129.6; 149.0; 153.7; 165.9; 166.3. Mass spectrum *m/z* (ESI, %): 323 $[M+H]^+$ (100); 324 (15), 325 (32). Found, %: C 48.29; H 3.38; N 17.51. $C_{13}H_{11}ClN_4O_4$. Calculated, %: C 48.39; H 3.44; N 17.36.

General procedure for the synthesis of 5-aryl-2-(methylthio)-7-((3-(methylthio)-1*H*-1,2,4-triazol-5-yl)amino)-4,5,6,7-tetrahydro-[1,2,4]triazolo[1,5-*a*]pyrimidine-7-carboxylic acid 5a–c
A mixture of 5-amino-3-methylthio-1*H*-1,2,4-triazole (**1a**, 130 mg, 1 mmol), salicylaldehydes **2a,c,f** (0,5 mmol) and pyruvic acid (**3**, 44 mg, 0,5 mmol) in 2 mL of acetic acid was stirred at room temperature for 72 h. The precipitate was filtered and dried, avoiding heating.

5-(2-Hydroxyphenyl)-2-(methylthio)-7-((3-(methylthio)-1*H*-1,2,4-triazol-5-yl)amino)-4,5,6,7-tetrahydro-[1,2,4]triazolo[1,5-*a*]pyrimidine-7-carboxylic acid (5a)

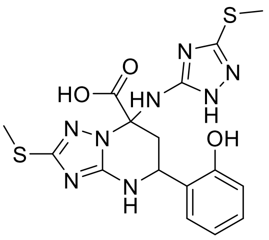

White solid (125 mg, 58%), a mixture of diastereomers in the ratio (60:40). ^{1}H NMR spectrum, (400 MHz, DMSO-*d*$_6$), δ, ppm (*J*, Hz): 2.25-2.34 (1H, m, CH_2); 2.37-2.43 (3H, s, CH_3S); 2.88-2.98 (0.4H, m, CH_2); 3.01-3.11 (0.6H, m, CH_2); 5.05-5.10 (0.6H, m, CH); 5.11-5.14 (0.4H, m, CH); 6.80-7.39 (4H, m, ArH); 7.46 (0.6H, s, NH); 7.77 (0.4H, s, NH); 9.55 (0.4H, s, OH); 9.65 (0.6H, s, OH); 12.87 (1H, br s, COOH). ^{13}C NMR spectrum, (100 MHz, DMSO-*d*$_6$), δ, ppm: 13.5; 13.6; 13.8; 36.9; 37,1; 45.2; 46.5; 72,0; 72.3; 115.1; 119.2; 119.3; 126.4; 126.8; 126.9; 127.1; 128.4; 154.12; 154.5; 1568.1; 156.9; 157.2; 158.3; 169.0; 170.2. Mass spectrum *m/z* (ESI, %): 435 $[M+H]^+$ (100); 436 (11), 271 (18), 235 (44). Found, %: C 44.14; H 4.06; N 25.87. $C_{16}H_{18}N_8O_3S_2$. Calculated, %: C 44.23; H 4.18; N 25.79.

5-(5-Bromo-2-hydroxyphenyl)-2-(methylthio)-7-((3-(methylthio)-1*H*-1,2,4-triazol-5-yl)amino)-4,5,6,7-tetrahydro-[1,2,4]triazolo[1,5-*a*]pyrimidine-7-carboxylic acid (5b)

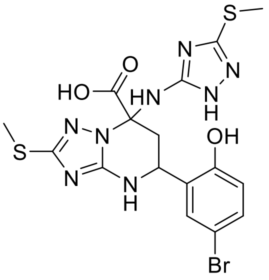

White solid (175 mg, 69%), a mixture of diastereomers in the ratio (50:50). ^{1}H NMR spectrum, (400 MHz, DMSO-*d*$_6$), δ, ppm (*J*, Hz): 2.28-2.37 (1H, m, CH_2); 2.41 -2.44 (3H, s, CH_3S); 2.95-3.11 (1H, m, CH_2); 5.01-5.06 (0.5H, m, CH); 5.08-5.13 (0.5H, m, CH); 6.74-7.51 (3H, m, ArH): 7.53 (0.5H, s, NH); 7.84 (0.5H, s, NH); 9.93 (0.5H, s, OH); 10.05 (0.5H, s, OH); 12.80 (1H, br s, COOH). ^{13}C NMR spectrum, (100 MHz, DMSO-*d*$_6$), δ, ppm: 13.5; 13.6; 13.7; 13.8; 36.4; 36.8; 45.2; 46.3; 71.8; 72.0; 110.3; 110.4; 117.3; 117.4; 129.1; 129.5; 129.9; 130.9; 130.8; 153.6; 153.8; 155.9; 156.7; 157.3;

158.3; 168.9; 170.0;. Mass spectrum *m/z* (ESI, %): 513 $[M+H]^+$ (91), 315 (77), 313 (69), 201 (26). Found, %: C 37.24; H 3.26; N 21.87. $C_{16}H_{17}BrN_8O_3S_2$. Calculated, %: C 37.43; H 3.34; N 21.83.

5-(5-Chloro-2-hydroxyphenyl)-2-(methylthio)-7-((3-(methylthio)-1*H*-1,2,4-triazol-5-yl)amino)-4,5,6,7-tetrahydro-[1,2,4]triazolo[1,5-*a*]pyrimidine-7-carboxylic acid (5c)

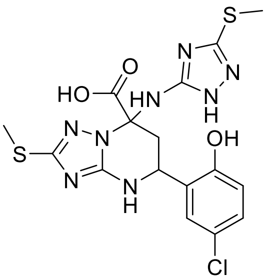

White solid (155 mg, 67%), a mixture of diastereomers in the ratio (50:50). ^{1}H NMR spectrum, (400 MHz, DMSO-d_6), δ, ppm (*J*, Hz): 2.29-2.37 (1H, m, CH_2); 2.37-2.43 (3H, s, CH_3S); 2.94-2.99 (0.5H, m, CH_2); 3.06-3.11 (0.5H, m, CH_2); 4.98-5.06 (0.5H, m, CH); 5.08-5.13 (0.5H, m, CH); 6.78-7.37 (3H, m, ArH): 7.54 (0.5H, s, NH); 7.84 (0.5H, s, NH); 9.90 (0.5H, s, OH); 10.02 (0.5H, s, OH); 12.95 (1H, br s, COOH). ^{13}C NMR spectrum, (100 MHz, DMSO-d_6), δ, ppm: 13.4; 13.5; 13.8; 36.3; 36.8, 45.2; 46.3; 71.8; 72.0; 116.7; 116.8; 122.6; 122.8; 126.2; 126.7; 128.0; 128.9; 129.4; 153.1; 153.4; 156.0; 156.7; 157.3; 158.2; 168.9; 170.0. Mass spectrum *m/z* (ESI, %): 469 $[M+H]^+$ (100); 471 (44), 271 (18), 269 (48). Found, %: C 40.91; H 3.69; N 23.97. $C_{16}H_{17}ClN_8O_3S_2$. Calculated, %: C 40.98; H 3.65; N 23.90.

Copies of ^{1}H, and ^{13}C NMR spectra

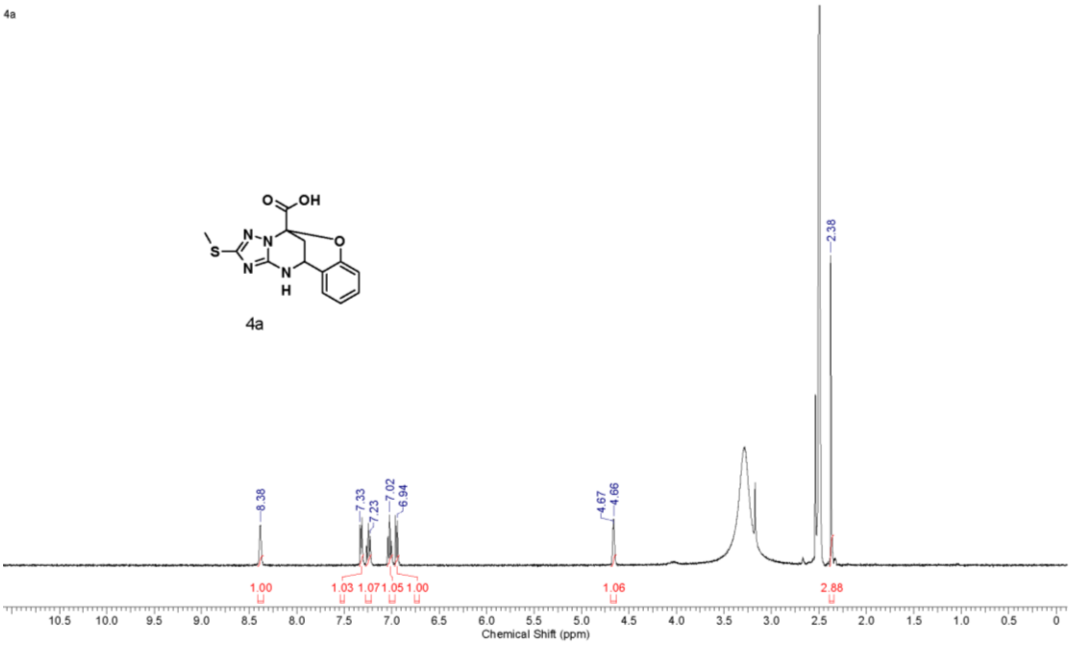

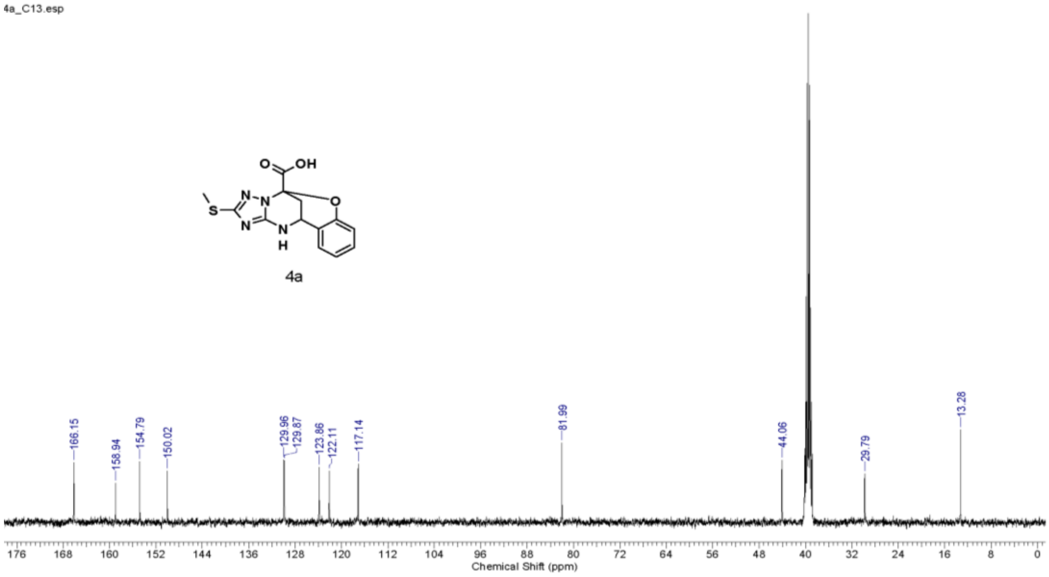

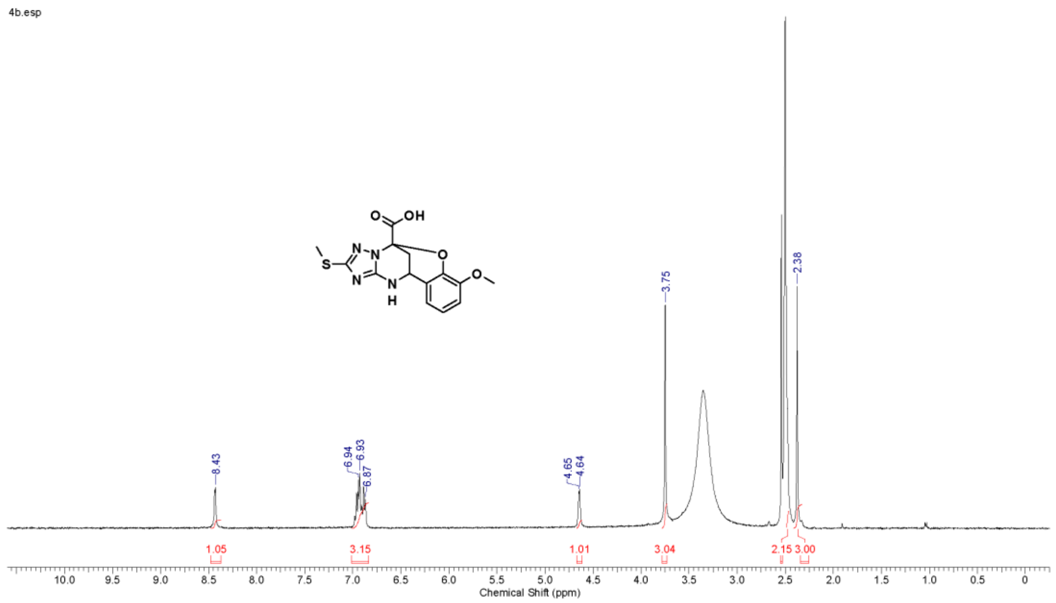
4b.esp
8.43
6.94
6.93
6.87
4.65
4.64
3.75
2.38
1.05
3.15
1.01
3.04
2.15
3.00
Chemical Shift (ppm)

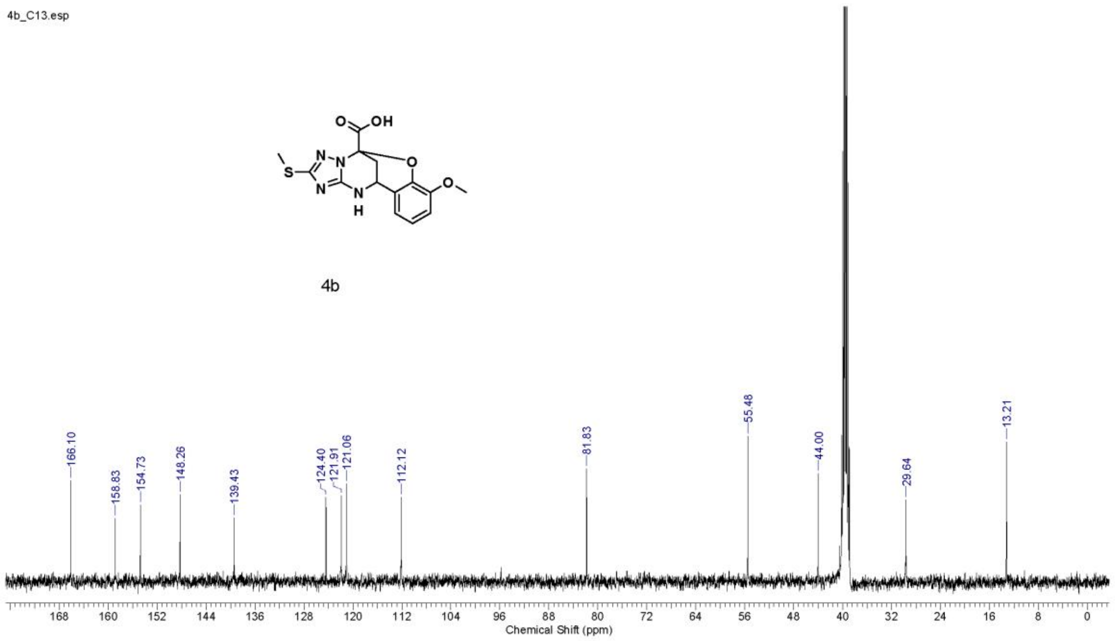
4b_C13.esp
4b
166.10
158.83
154.73
148.26
139.43
124.40
121.91
121.06
112.12
81.83
55.48
44.00
29.64
13.21
Chemical Shift (ppm)

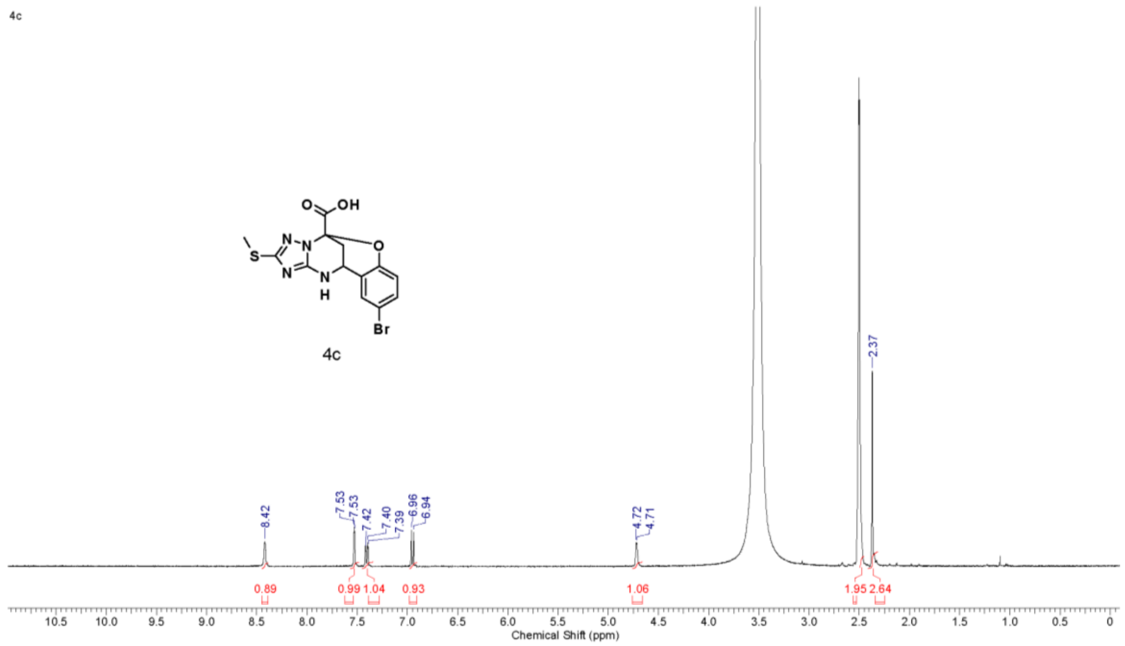
4c
O OH
S
N N N
N
N H
O
Br
4c
8.42
7.53
7.53
7.42
7.40
7.39
6.96
6.94
4.72
4.71
2.37
0.89
0.99
1.04
0.93
1.06
1.95
2.64
10.5 10.0 9.5 9.0 8.5 8.0 7.5 7.0 6.5 6.0 5.5 5.0 4.5 4.0 3.5 3.0 2.5 2.0 1.5 1.0 0.5 0
Chemical Shift (ppm)

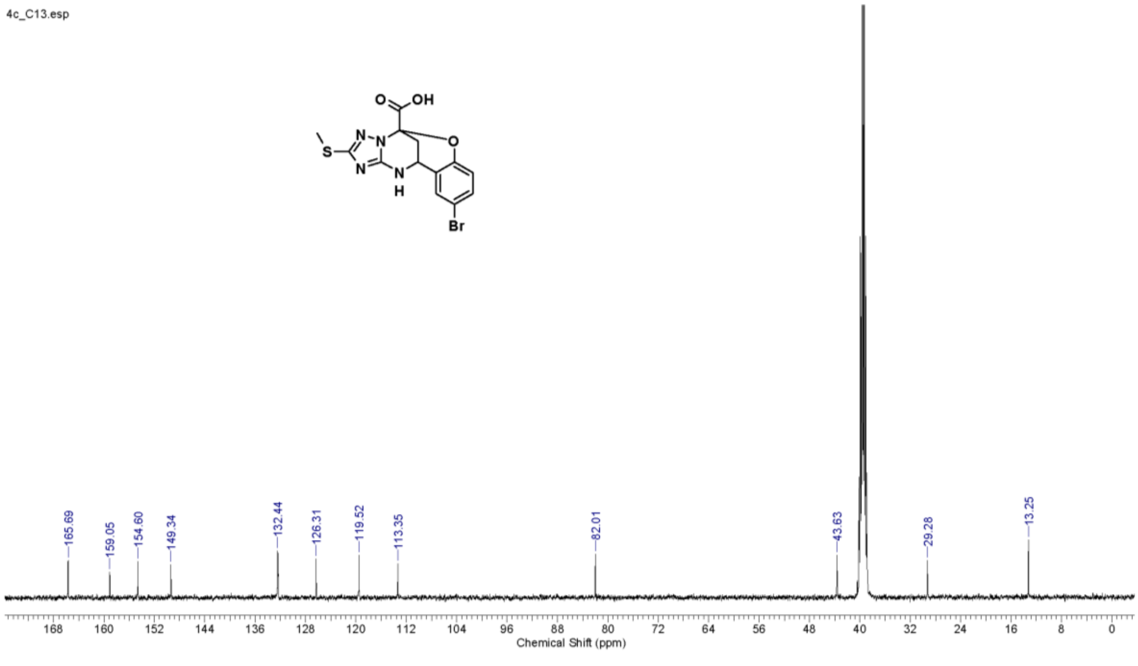
4c_C13.esp
O OH
S
N N N
N
N H
O
Br
165.69
159.05
154.60
149.34
132.44
126.31
119.52
113.35
82.01
43.63
29.28
13.25
168 160 152 144 136 128 120 112 104 96 88 80 72 64 56 48 40 32 24 16 8 0
Chemical Shift (ppm)

4d

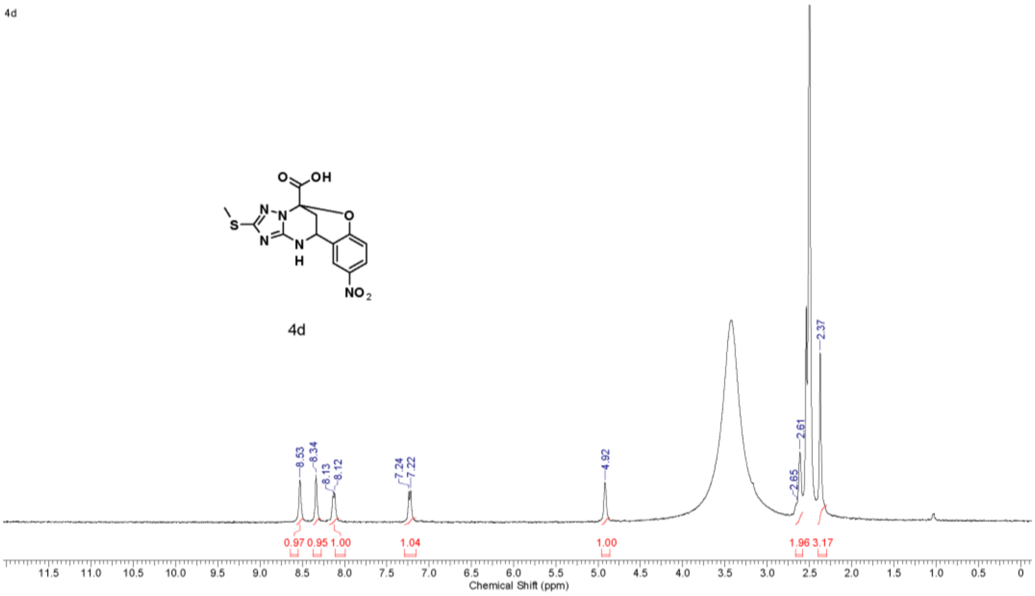

COOH
S
N
H
NO2
4d
8.53
8.34
8.13
8.12
7.24
7.22
4.92
2.65
2.61
2.37
0.97
0.95
1.00
1.04
1.00
1.96
3.17
11.5 11.0 10.5 10.0 9.5 9.0 8.5 8.0 7.5 7.0 6.5 6.0 5.5 5.0 4.5 4.0 3.5 3.0 2.5 2.0 1.5 1.0 0.5 0
Chemical Shift (ppm)

4d_C13.esp

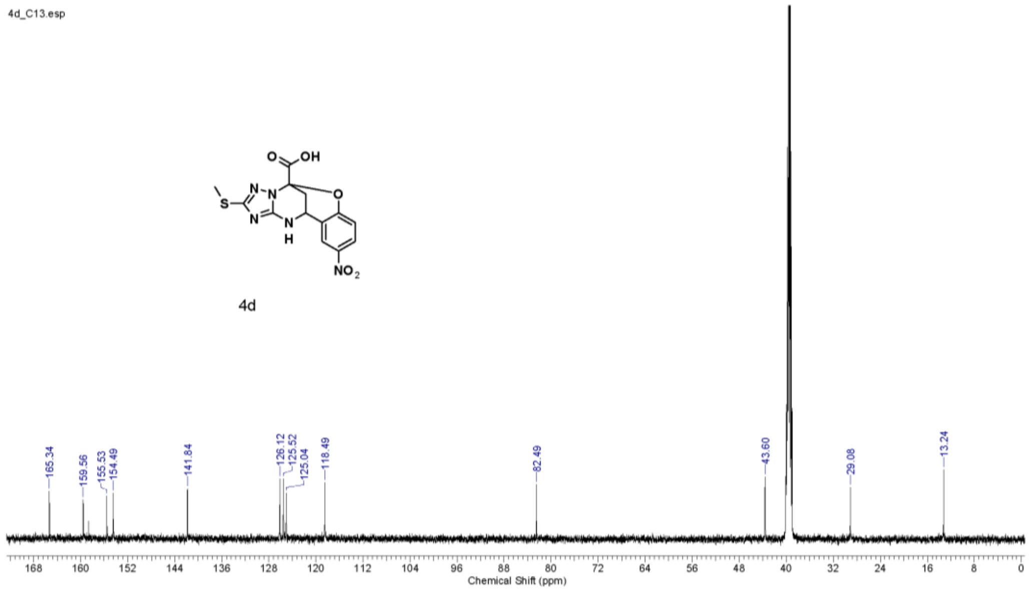

COOH
S
N
H
NO2
4d
165.34
159.56
155.53
154.49
141.84
126.12
125.52
125.04
118.49
82.49
43.60
29.08
13.24
168 160 152 144 136 128 120 112 104 96 88 80 72 64 56 48 40 32 24 16 8 0
Chemical Shift (ppm)

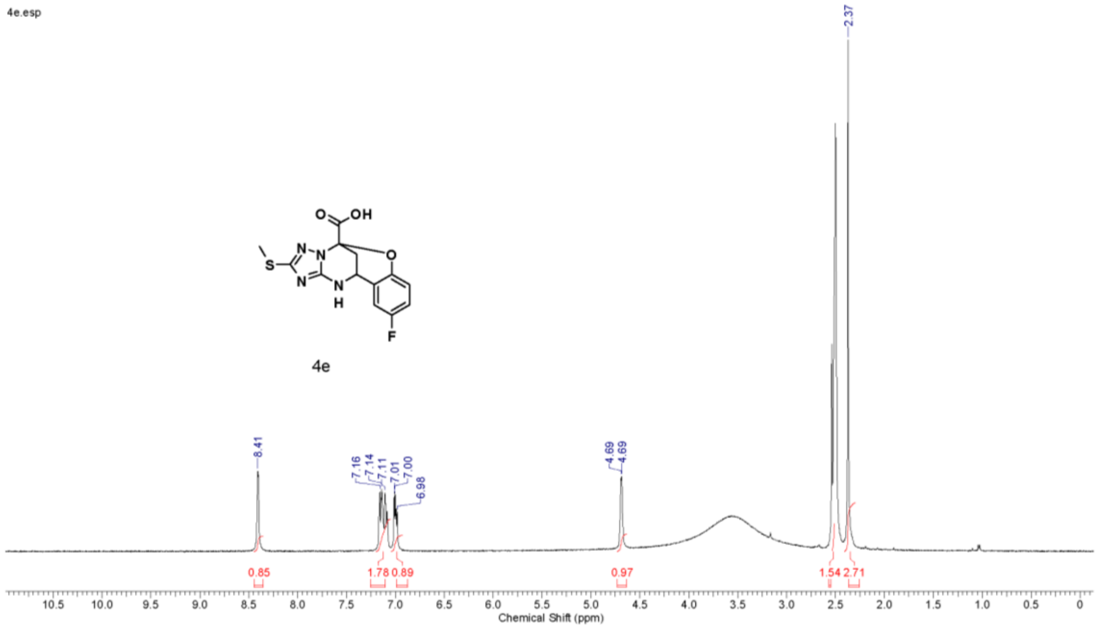

4e.esp
OH
O
N
N
N
S
N
H
O
F
4e
8.41
7.16
7.14
7.11
7.01
7.00
6.98
4.69
4.69
2.37
0.85
1.78
0.89
0.97
1.54
2.71
10.5 10.0 9.5 9.0 8.5 8.0 7.5 7.0 6.5 6.0 5.5 5.0 4.5 4.0 3.5 3.0 2.5 2.0 1.5 1.0 0.5 0
Chemical Shift (ppm)

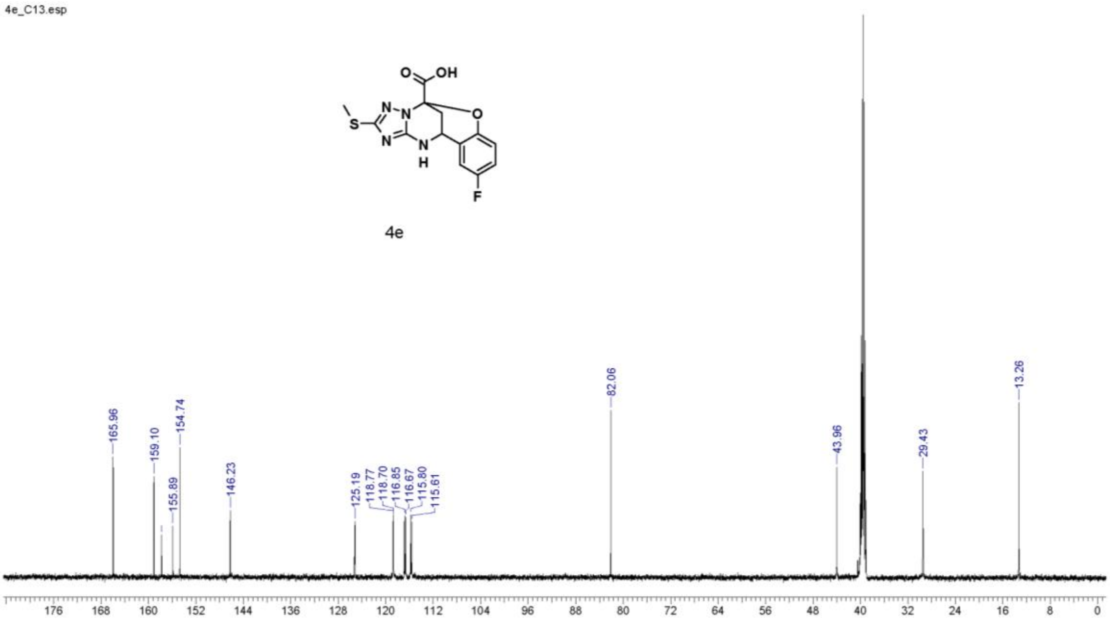

4e_C13.esp
OH
O
N
N
N
S
N
H
O
F
4e
165.96
159.10
155.89
154.74
146.23
125.19
118.77
118.70
116.85
116.67
115.80
115.61
82.06
43.96
29.43
13.26
176 168 160 152 144 136 128 120 112 104 96 88 80 72 64 56 48 40 32 24 16 8 0

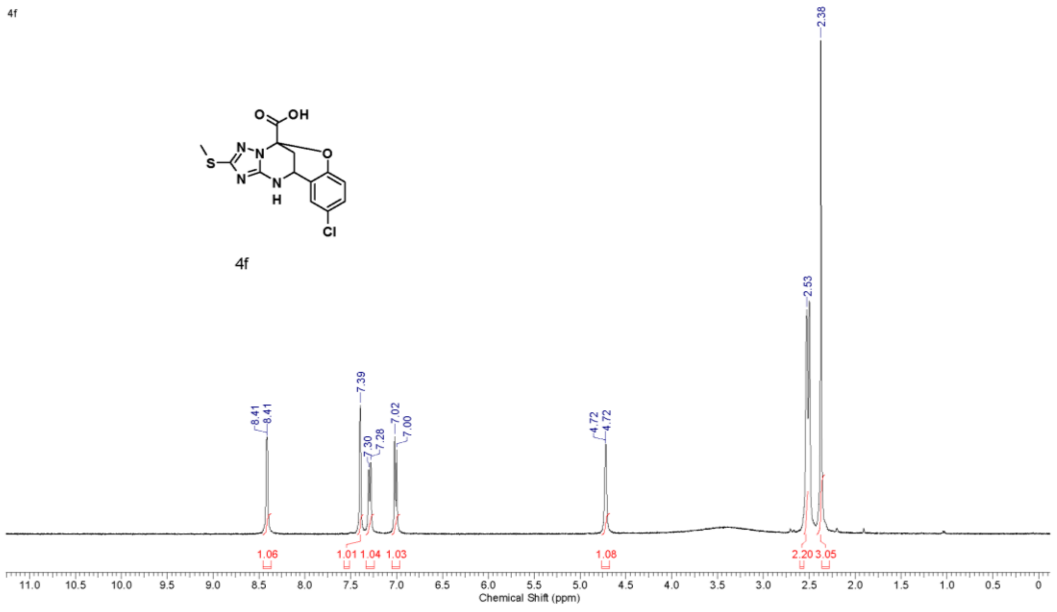
4f
O OH
N N S N N H O Cl
4f
8.41
8.41
7.39
7.30
7.28
7.02
7.00
4.72
4.72
2.53
2.38
1.06
1.01
1.04
1.03
1.08
2.20
3.05
11.0 10.5 10.0 9.5 9.0 8.5 8.0 7.5 7.0 6.5 6.0 5.5 5.0 4.5 4.0 3.5 3.0 2.5 2.0 1.5 1.0 0.5 0
Chemical Shift (ppm)

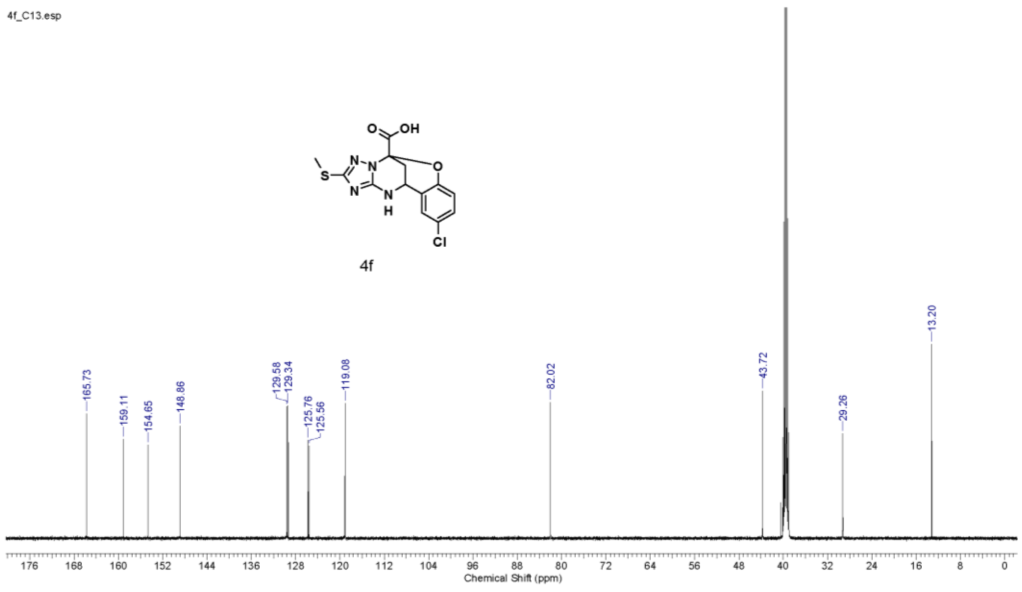
4f_C13.esp
O OH
N N S N N H O Cl
4f
165.73
159.11
154.65
148.86
129.58
129.34
125.76
125.56
119.08
82.02
43.72
29.26
13.20
176 168 160 152 144 136 128 120 112 104 96 88 80 72 64 56 48 40 32 24 16 8 0
Chemical Shift (ppm)

g

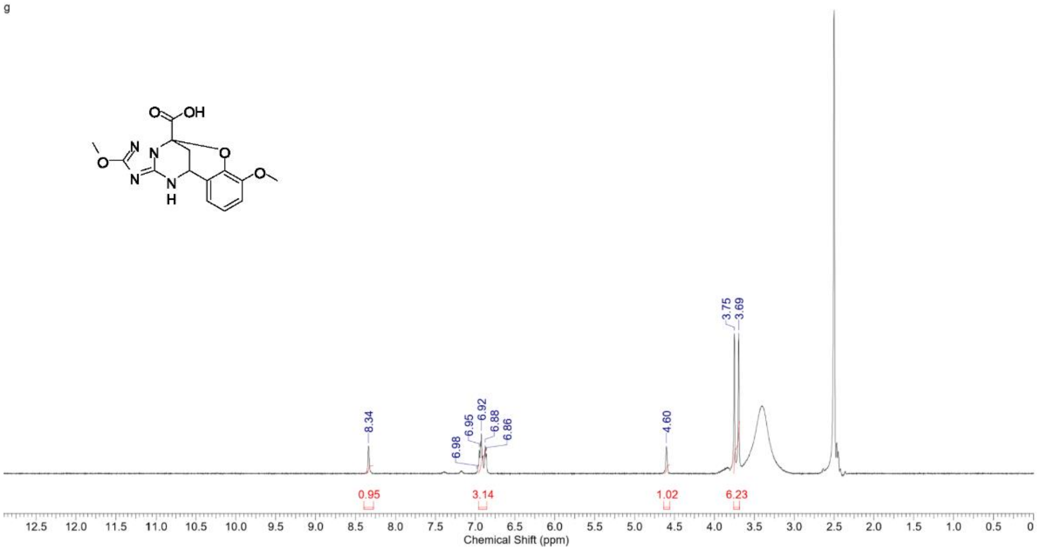

OH
O
N
N
N
H
O
O
8.34
6.98
6.95
6.92
6.88
6.86
4.60
3.75
3.69
0.95
3.14
1.02
6.23
12.5 12.0 11.5 11.0 10.5 10.0 9.5 9.0 8.5 8.0 7.5 7.0 6.5 6.0 5.5 5.0 4.5 4.0 3.5 3.0 2.5 2.0 1.5 1.0 0.5 0
Chemical Shift (ppm)

4g_C13

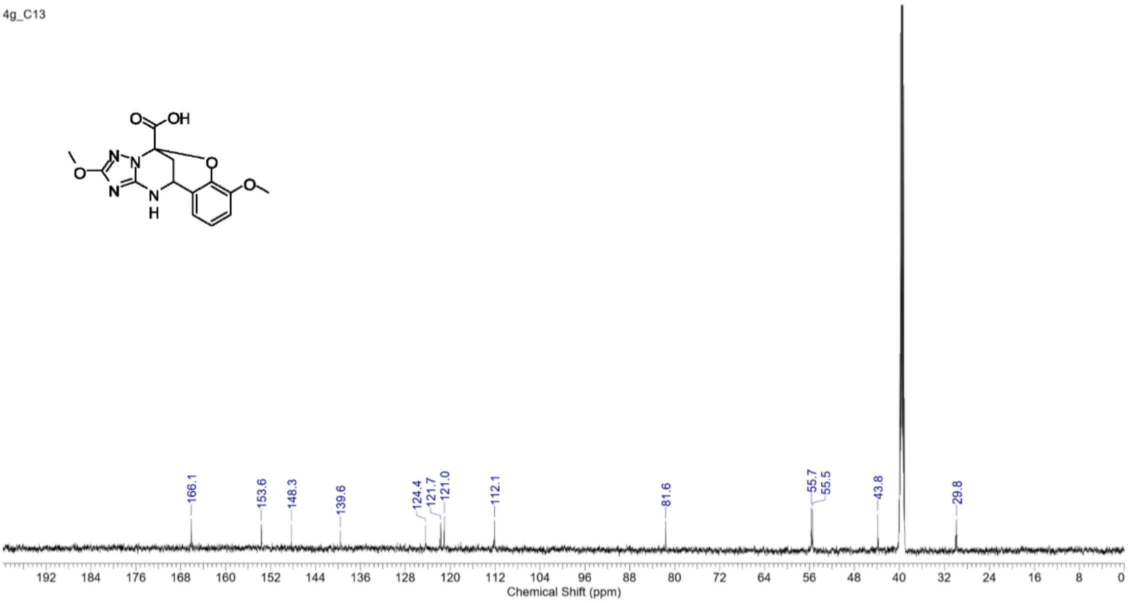

OH
O
N
N
N
H
O
O
166.1
153.6
148.3
139.6
124.4
121.7
121.0
112.1
81.6
55.7
55.5
43.8
29.8
192 184 176 168 160 152 144 136 128 120 112 104 96 88 80 72 64 56 48 40 32 24 16 8 0
Chemical Shift (ppm)

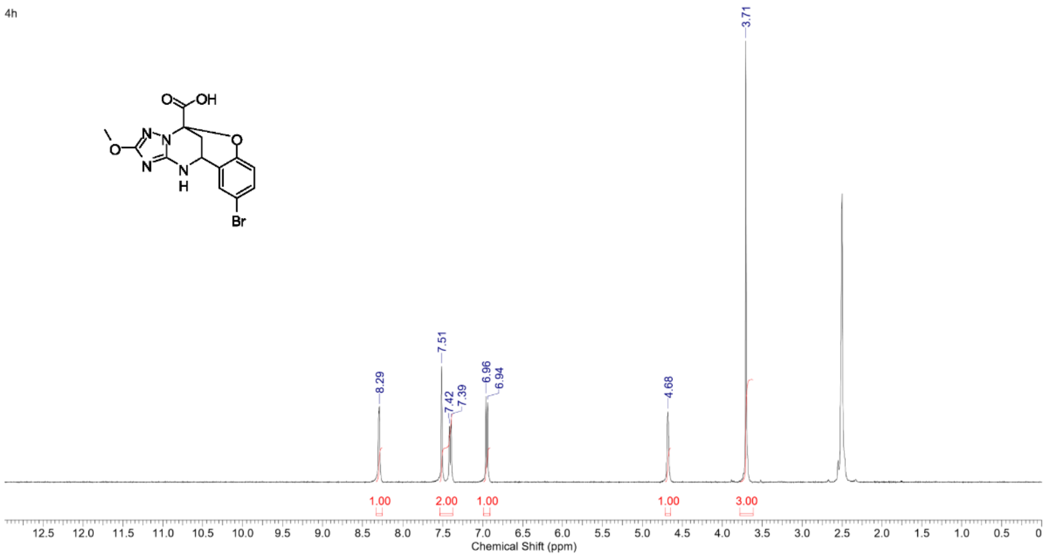
4h
3.71
8.29
7.51
7.42
7.39
6.96
6.94
4.68
1.00
2.00
1.00
1.00
3.00
12.5 12.0 11.5 11.0 10.5 10.0 9.5 9.0 8.5 8.0 7.5 7.0 6.5 6.0 5.5 5.0 4.5 4.0 3.5 3.0 2.5 2.0 1.5 1.0 0.5 0
Chemical Shift (ppm)

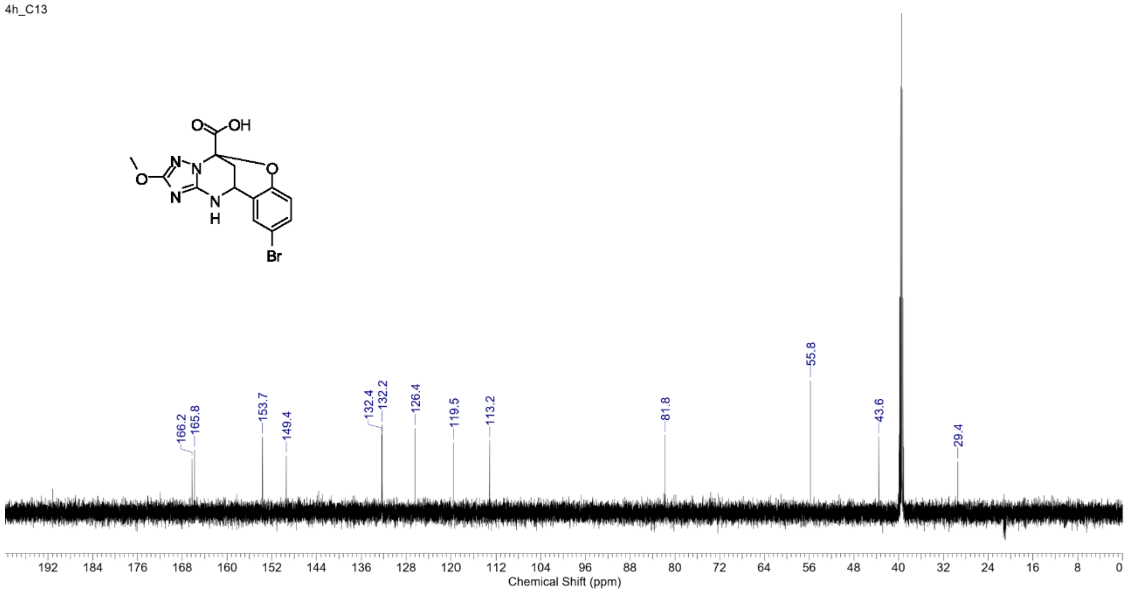
4h_C13
166.2
165.8
153.7
149.4
132.4
132.2
126.4
119.5
113.2
81.8
55.8
43.6
29.4
192 184 176 168 160 152 144 136 128 120 112 104 96 88 80 72 64 56 48 40 32 24 16 8 0
Chemical Shift (ppm)

4i

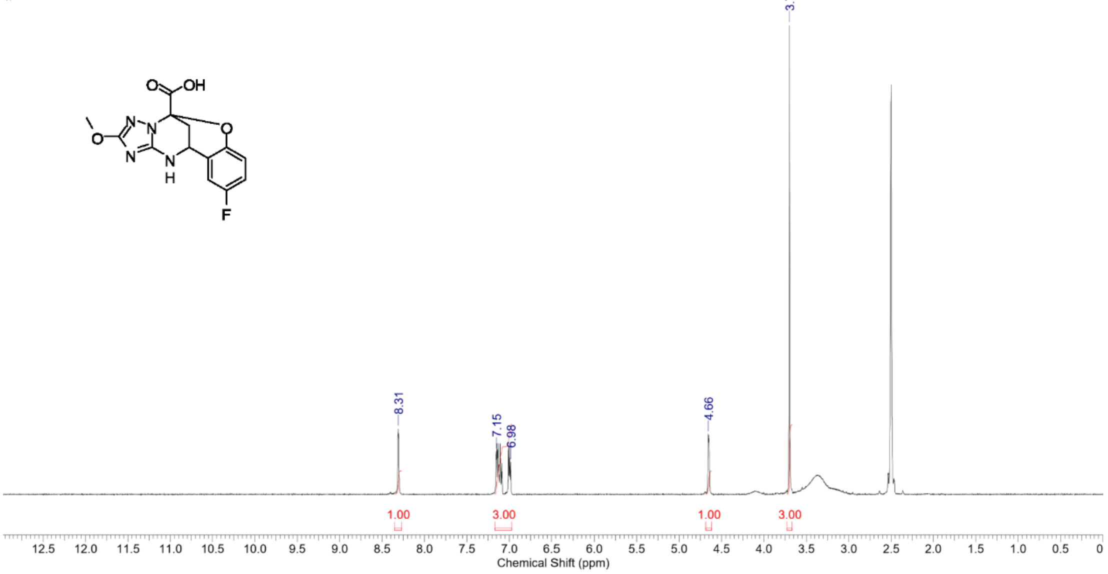

8.31
7.15
6.98
4.66
3.70
1.00
3.00
1.00
3.00
12.5 12.0 11.5 11.0 10.5 10.0 9.5 9.0 8.5 8.0 7.5 7.0 6.5 6.0 5.5 5.0 4.5 4.0 3.5 3.0 2.5 2.0 1.5 1.0 0.5 0
Chemical Shift (ppm)

4i_C13

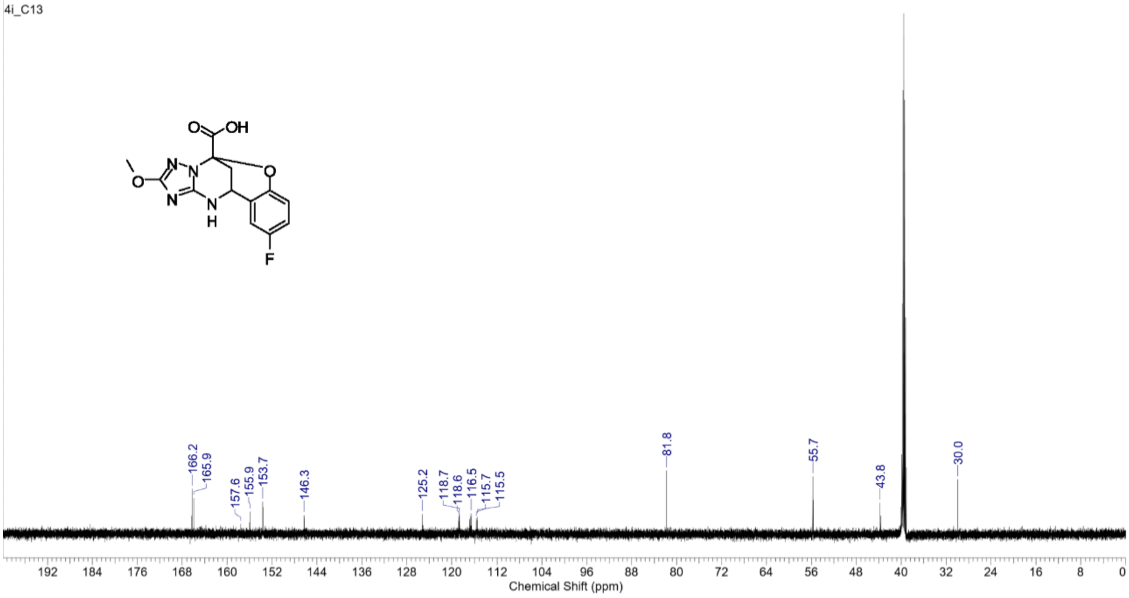

166.2
165.9
157.6
155.9
153.7
146.3
125.2
118.7
118.6
116.5
115.7
115.5
81.8
55.7
43.8
30.0
192 184 176 168 160 152 144 136 128 120 112 104 96 88 80 72 64 56 48 40 32 24 16 8 0
Chemical Shift (ppm)

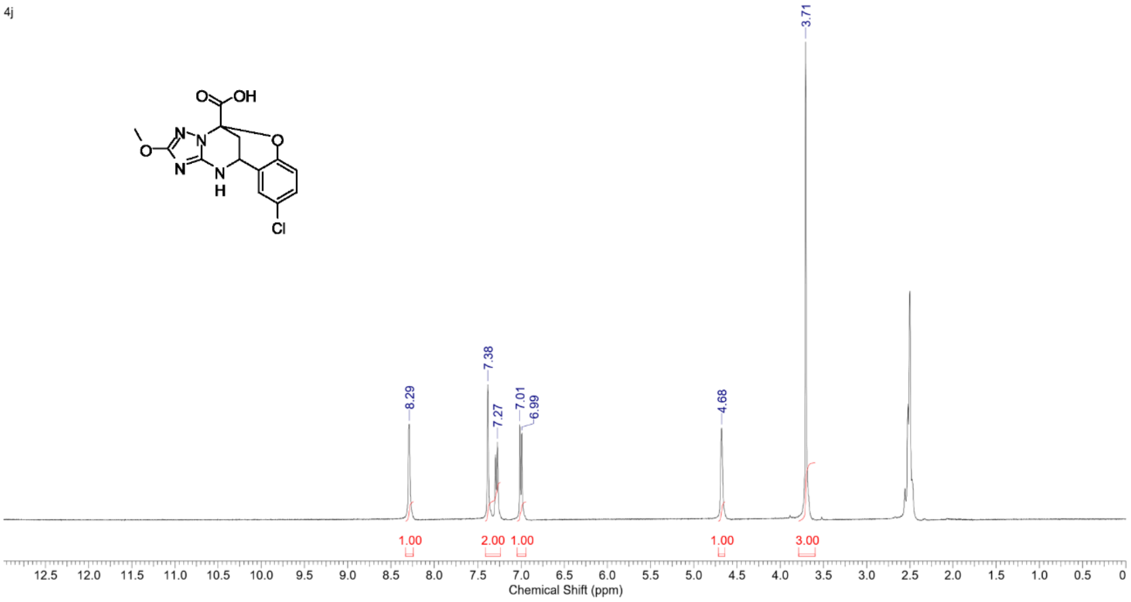
4j
OH
O
N
N
N
O
O
N
H
Cl
8.29
7.38
7.27
7.01
6.99
4.68
3.71
1.00
2.00
1.00
1.00
3.00
12.5 12.0 11.5 11.0 10.5 10.0 9.5 9.0 8.5 8.0 7.5 7.0 6.5 6.0 5.5 5.0 4.5 4.0 3.5 3.0 2.5 2.0 1.5 1.0 0.5 0
Chemical Shift (ppm)

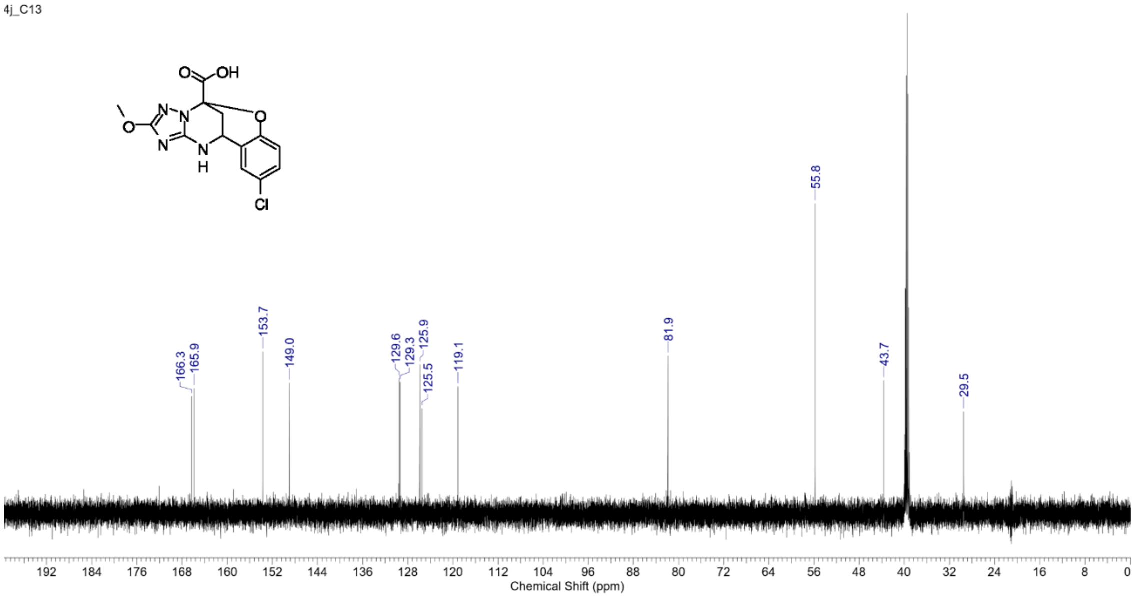
4j_C13
OH
O
N
N
N
O
O
N
H
Cl
166.3
165.9
153.7
149.0
129.6
129.3
125.9
125.5
119.1
81.9
55.8
43.7
29.5
192 184 176 168 160 152 144 136 128 120 112 104 96 88 80 72 64 56 48 40 32 24 16 8 0
Chemical Shift (ppm)

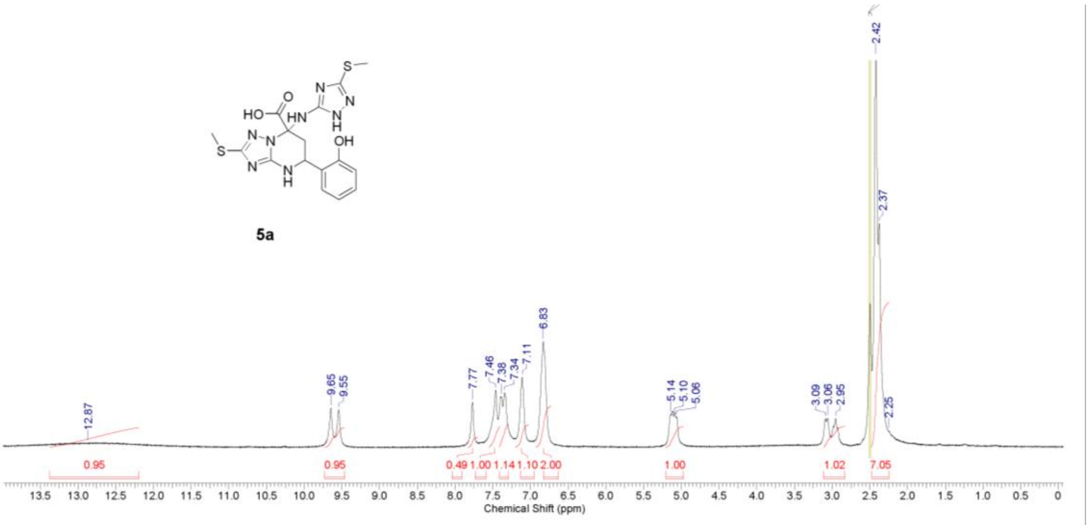
5a
12.87
9.65
9.55
7.77
7.46
7.38
7.34
7.11
6.83
5.14
5.10
5.06
3.09
3.06
2.95
2.42
2.37
2.25
0.95
0.95
0.49
1.00
1.14
1.10
2.00
1.00
1.02
7.05
Chemical Shift (ppm)

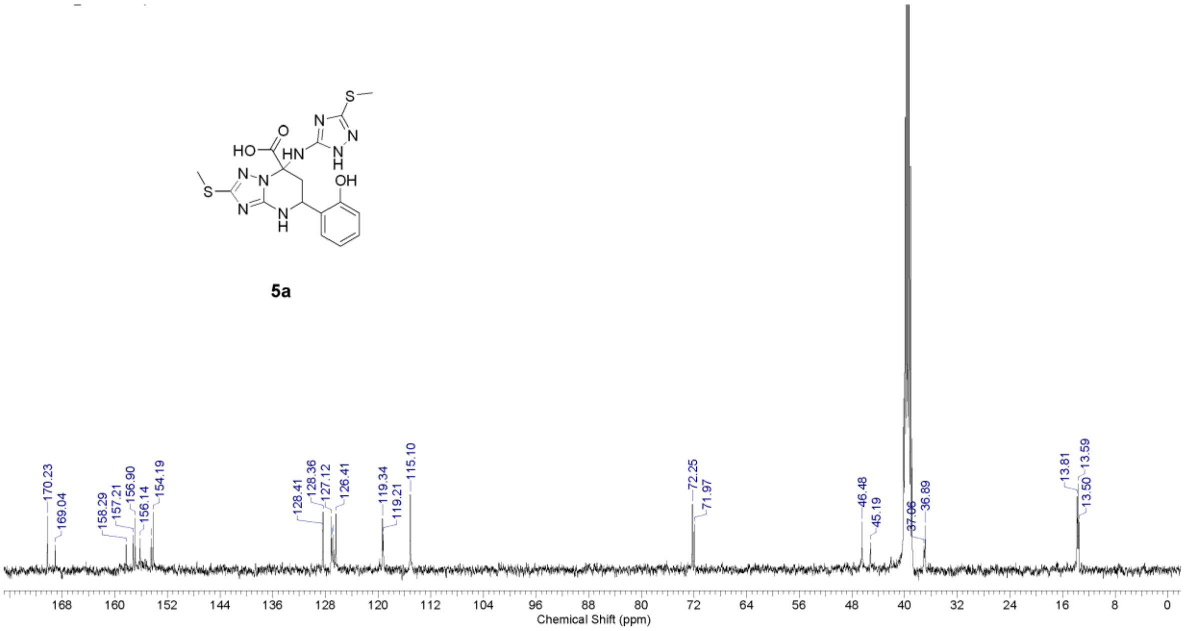
5a
170.23
169.04
158.29
157.21
156.90
156.14
154.19
128.41
128.36
127.12
126.41
119.34
119.21
115.10
72.25
71.97
46.48
45.19
37.06
36.89
13.81
13.59
13.50
Chemical Shift (ppm)

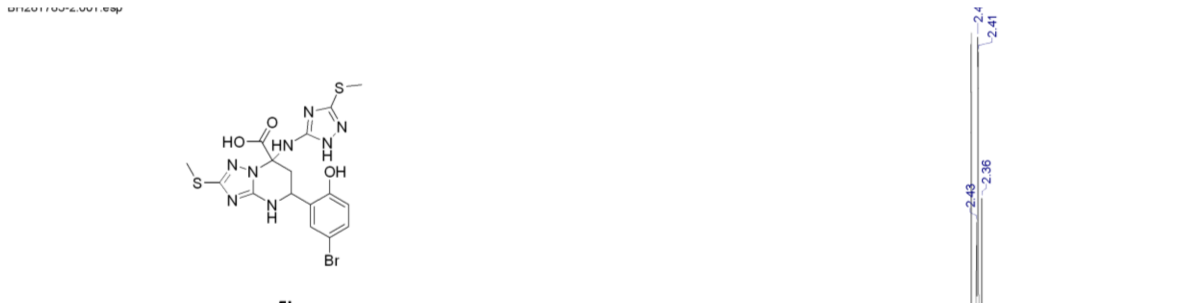

HO
O
HN
N
N
N
H
S
N
N
S
N
N
H
OH
Br
10.05
9.93
12.80
7.84
7.53
7.50
7.43
7.27
7.27
6.80
6.78
6.77
6.75
5.12
5.09
5.05
5.02
3.10
3.07
3.00
2.96
2.43
2.41
2.36
2.32
2.29
1.07
1.00
0.53
0.59
0.62
0.63
1.17
1.03
1.00
1.01
7.27
Chemical Shift (ppm)

5b

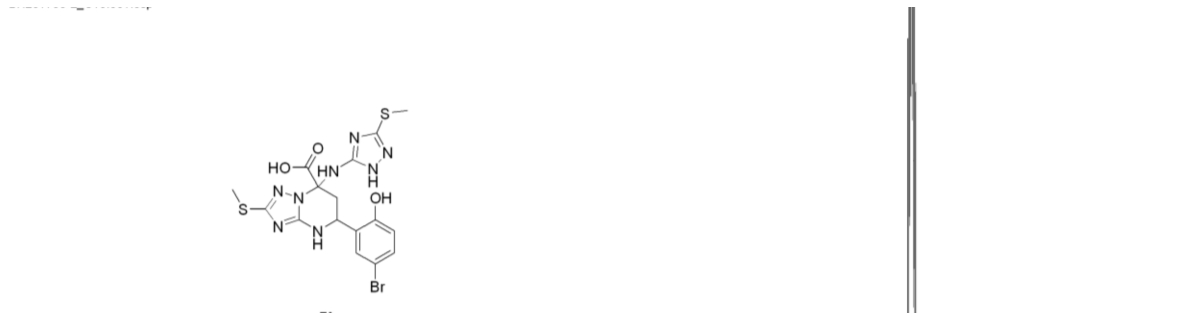

HO
O
HN
N
N
N
H
S
N
N
S
N
N
H
OH
Br
170.04
168.89
158.26
157.32
156.68
153.83
153.58
130.91
129.91
129.11
117.35
117.27
110.42
110.27
71.99
71.78
46.32
45.24
36.84
36.38
13.78
13.55
13.48
Chemical Shift (ppm)

5b

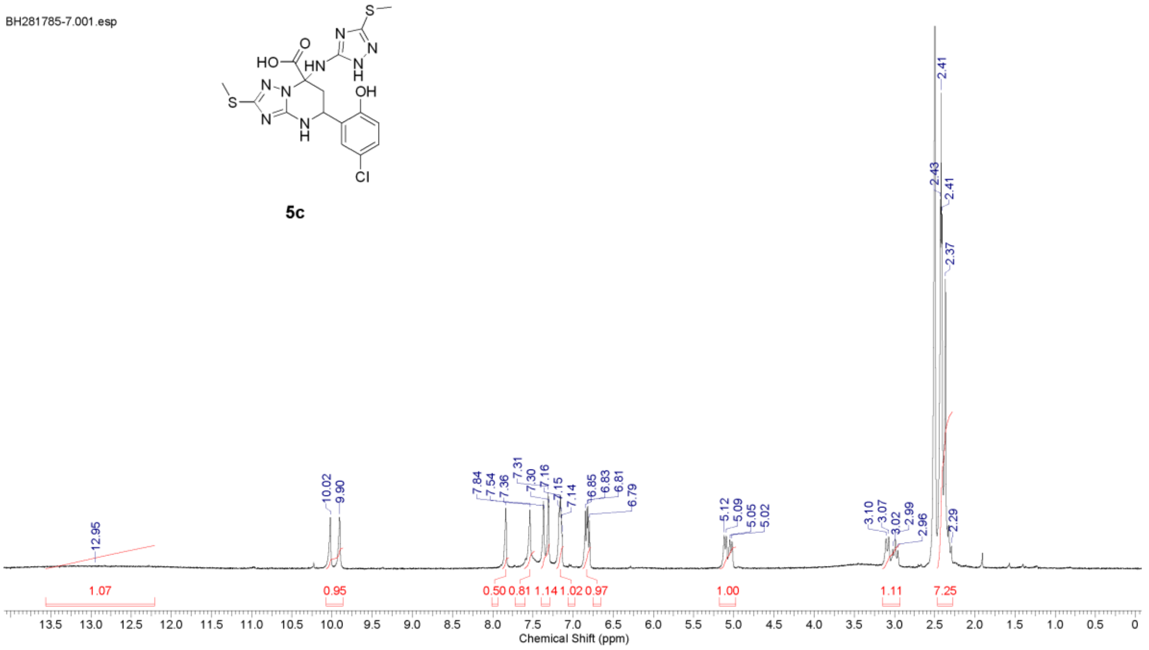
BH281785-7.001.esp
5c
12.95
10.02
9.90
7.84
7.54
7.36
7.31
7.30
7.16
7.15
7.14
6.85
6.83
6.81
6.79
5.12
5.09
5.05
5.02
3.10
3.07
3.02
2.99
2.96
2.43
2.41
2.41
2.37
2.29
1.07
0.95
0.50
0.81
1.14
1.02
0.97
1.00
1.11
7.25
Chemical Shift (ppm)

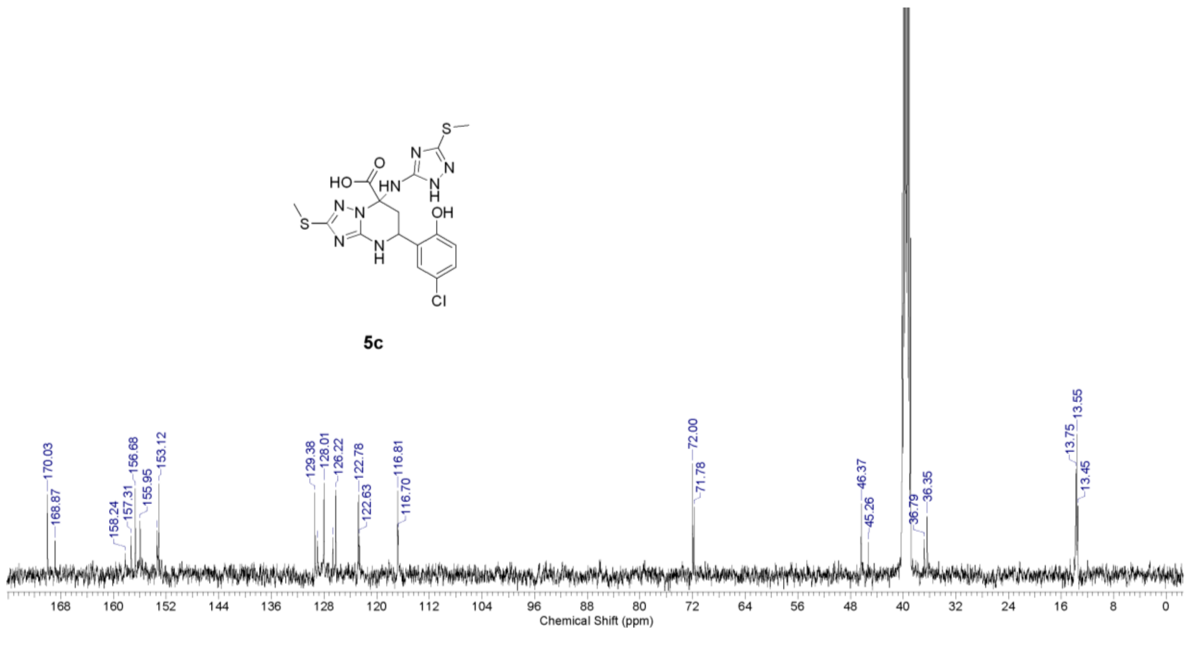
5c
170.03
168.87
158.24
157.31
156.68
155.95
153.12
129.38
128.01
126.22
122.78
122.63
116.81
116.70
72.00
71.78
46.37
45.26
36.79
36.35
13.75
13.55
13.45
Chemical Shift (ppm)

BH281785-1.001.esp

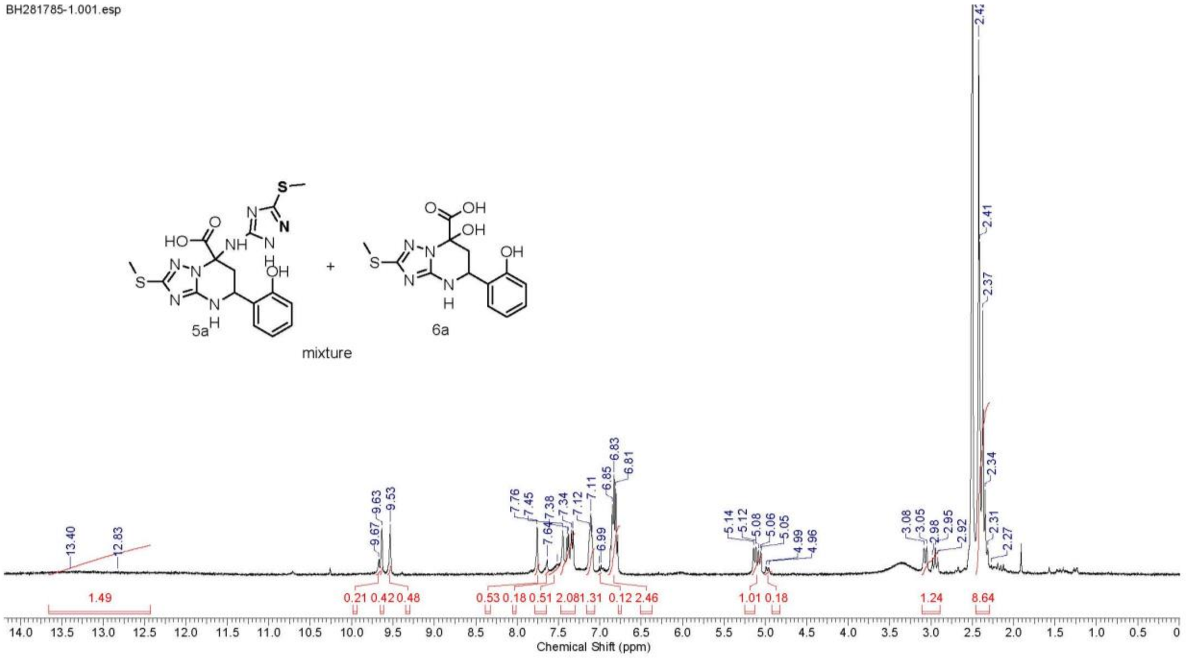

BH281785-4.001.esp

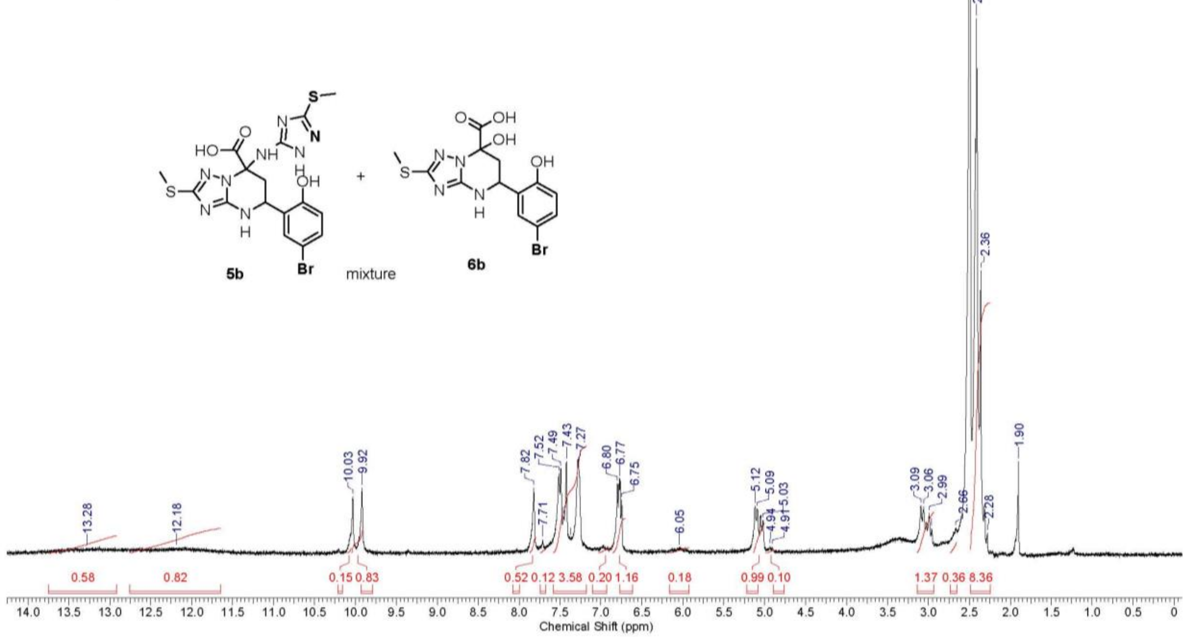

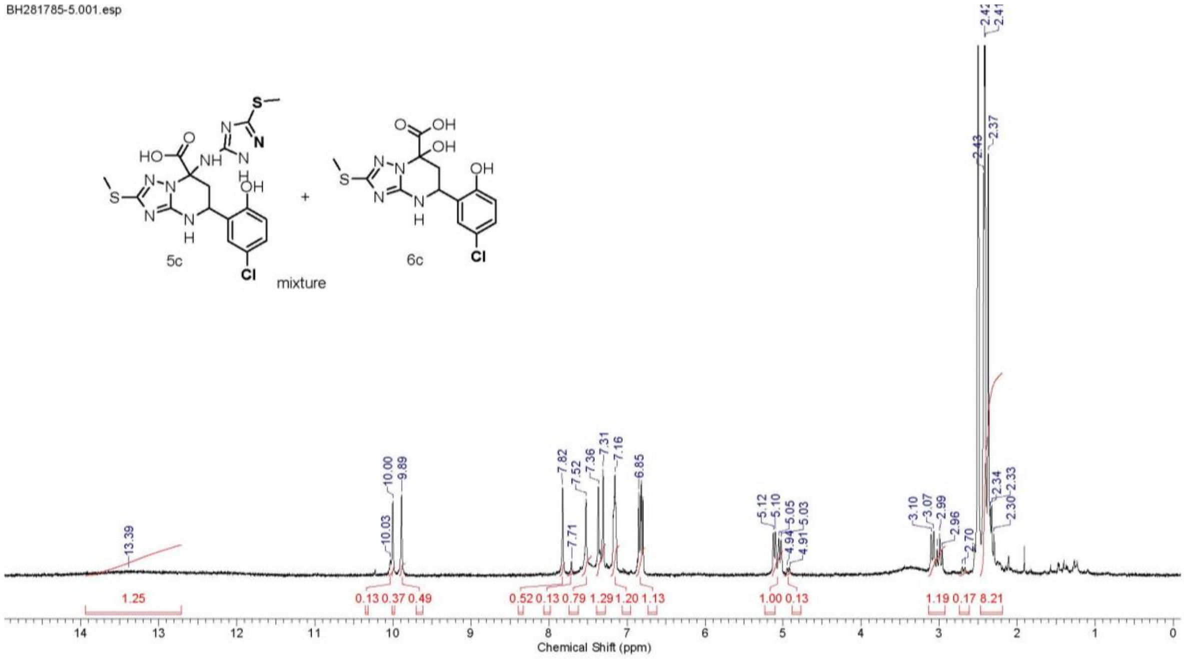
BH281785-5.001.esp
5c
6c
mixture
Chemical Shift (ppm)

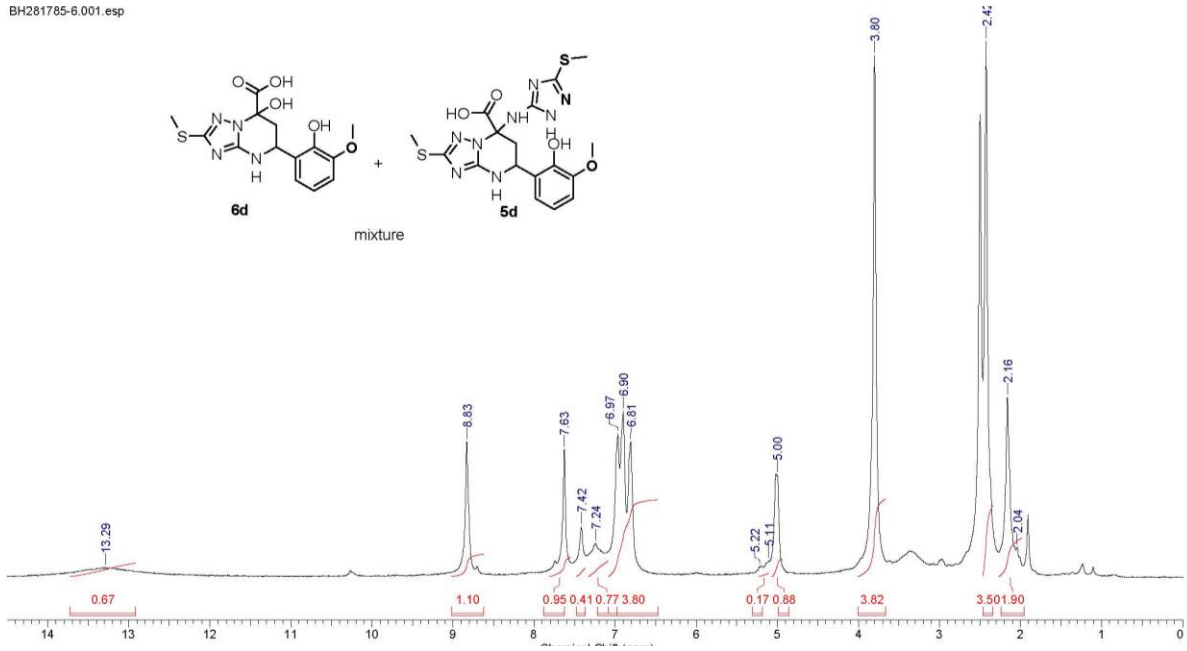
BH281785-6.001.esp
6d
5d
mixture

References

1. O. V. Dolomanov, L.J. Bourhis, R.J. Gildea, J.A.K. Howard, H. Puschmann, OLEX2 : a complete structure solution, refinement and analysis program, J. Appl. Crystallogr. 42 (2009) 339–341. https://doi.org/10.1107/S0021889808042726.
2. G.M. Sheldrick, SHELXT – Integrated space-group and crystal-structure determination, Acta Crystallogr. Sect. A Found. Adv. 71 (2015) 3–8. https://doi.org/10.1107/S2053273314026370.
3. G.M. Sheldrick, Crystal Structure Refinement with SHELXL, Acta Crystallogr. Sect. C, 71 (2015) 3–8. http://dx.doi.org/10.1107/S2053229614024218.
